# New Insights into the Interaction of Class II Dihydroorotate Dehydrogenases with Ubiquinone in Lipid Bilayers as a Function of Lipid Composition

**DOI:** 10.3390/ijms23052437

**Published:** 2022-02-23

**Authors:** Juan Manuel Orozco Rodriguez, Hanna P. Wacklin-Knecht, Luke A. Clifton, Oliver Bogojevic, Anna Leung, Giovanna Fragneto, Wolfgang Knecht

**Affiliations:** 1 Department of Biology & Lund Protein Production Platform, Lund University, Sölvegatan 35, 223 62 Lund, Sweden; manuel.orozco@biol.lu.se; 2 Department of Chemistry, Division of Physical Chemistry, Lund University, Naturvetarvägen 26, 222 41 Lund, Sweden; 3 European Spallation Source ERIC, P.O. Box 176, 221 00 Lund, Sweden; olbo@bce.au.dk (O.B.); anna.leung@ess.eu (A.L.); 4 ISIS Pulsed Neutron and Muon Source, Rutherford Appleton Laboratory, Didcot OX11 0QX, UK; luke.clifton@stfc.ac.uk; 5 Institut Laue-Langevin, 71 Avenue des Martyrs, BP 156, 38042 Grenoble, France; fragneto@ill.fr

**Keywords:** pyrimidine biosynthesis, protein–lipid interactions, membrane proteins, neutron reflectometry, ubiquinone

## Abstract

The fourth enzymatic reaction in the de novo pyrimidine biosynthesis, the oxidation of dihydroorotate to orotate, is catalyzed by dihydroorotate dehydrogenase (DHODH). Enzymes belonging to the DHODH Class II are membrane-bound proteins that use ubiquinones as their electron acceptors. We have designed this study to understand the interaction of an N-terminally truncated human DHODH (*Hs*Δ29DHODH) and the DHODH from *Escherichia coli* (*Ec*DHODH) with ubiquinone (Q_10_) in supported lipid membranes using neutron reflectometry (NR). NR has allowed us to determine in situ, under solution conditions, how the enzymes bind to lipid membranes and to unambiguously resolve the location of Q_10_. Q_10_ is exclusively located at the center of all of the lipid bilayers investigated, and upon binding, both of the DHODHs penetrate into the hydrophobic region of the outer lipid leaflet towards the Q_10_. We therefore show that the interaction between the soluble enzymes and the membrane-embedded Q_10_ is mediated by enzyme penetration. We can also show that *Ec*DHODH binds more efficiently to the surface of simple bilayers consisting of 1-palmitoyl, 2-oleoyl phosphatidylcholine, and tetraoleoyl cardiolipin than *Hs*Δ29DHODH, but does not penetrate into the lipids to the same degree. Our results also highlight the importance of Q_10_, as well as lipid composition, on enzyme binding.

## 1. Introduction

There are six enzymatic steps involved in the de novo pyrimidine biosynthesis pathway, which is nearly universal to all organisms [1,2,3,4]. The end product of the pathway, uridine monophosphate, is the starting point for the delivery of deoxynucleoside triphosphate (dNTP) precursors for the synthesis of DNA, nucleoside triphosphate (NTP) precursors for RNA, as well as glycoconjugates, and many more metabolically important molecules. The fourth enzymatic reaction in this pathway, the oxidation of dihydroorotate to orotate, is catalyzed by the flavoenzyme dihydroorotate dehydrogenase (DHODH) [5,6,7].

DHODHs can be divided into two major families or classes, I and II, based on the sequence similarities, rather than convergent evolution of different ancestral proteins, with a further subdivision of Class I into sub-classes IA and IB [8,9]. This division correlates with the quaternary structure, subcellular location of the enzymes, as well as their preferences for electron acceptors. Members of Class I are cytosolic, with Class IA enzymes being homodimers and Class IB enzymes being heterotetrameric proteins composed of two different proteins. DHODH from *Escherichia coli* is regarded as the prototype of Class II DHODHs [8,10]. In contrast to Class I enzymes, Class II DHODHs are monomeric membrane-bound proteins that use ubiquinones as electron receptors. 

Human DHODH (*Hs*DHODH) is located on the outside of the inner mitochondrial membrane (IMM) and uses ubiquinone Q_10_ as an electron acceptor, which functionally connects its activity to the respiratory chain [11,12,13]. Literature suggests that *Hs*DHODH is a stand-alone enzyme not associated with any respiratory supercomplexes [14]. *Hs*DHODH is probably the most studied member of the Class II enzymes because it is a target for anti-inflammatory drugs, such as leflunomide (ARAVA^®^), approved for rheumatoid arthritis and its active metabolite, and teriflunomide (AUBAGIO^®^), approved for multiple sclerosis, both proposed to interact with the same region of the enzyme as ubiquinone [15]. It was also recently validated as a target for the treatment of acute myeloid leukemia (AML) [16], as its inhibition overcomes the myeloid cell differentiation blockade. Therefore, novel and old *Hs*DHODH inhibitors are of interest for the treatment of hematological malignancies and innovative treatment options [17,18], and four compounds have entered clinical trials [19,20]. Furthermore, mutations in *Hs*DHODH have been identified as the cause of Miller syndrome [21,22], a rare autosomal recessive disorder (OMIM %263750) resulting in numerous abnormalities of the head, face, and limbs. *Hs*DHODH was also recently identified as the mitochondrial gatekeeper of cell death by ferroptosis because its deletion promotes ferroptosis [23]. 

**Figure 1:**
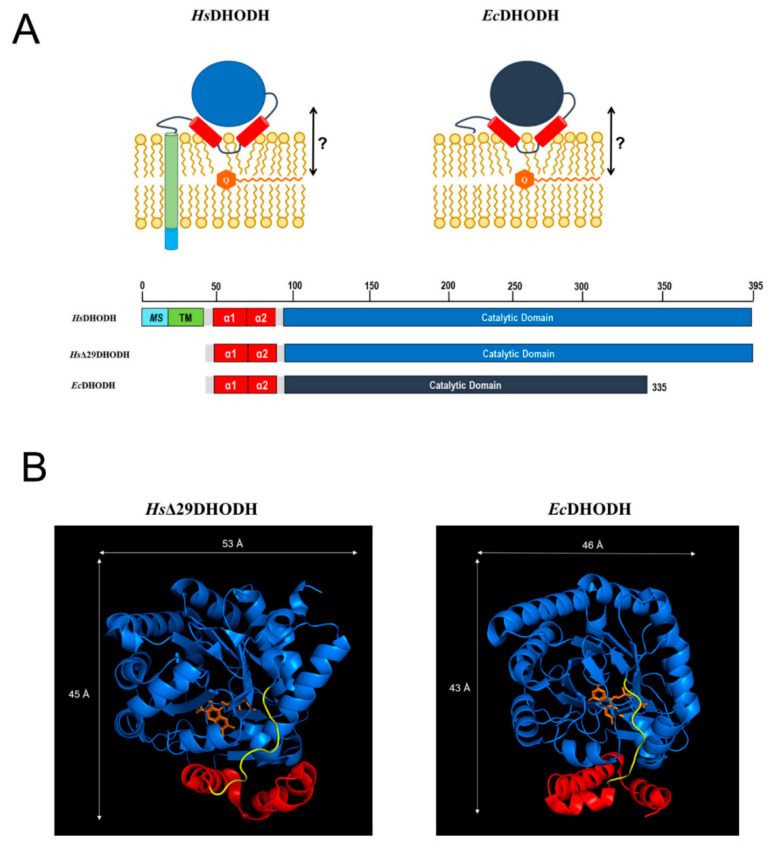
(**A**) Schematic representation of *Hs*DHODH and *Ec*DHODH in a lipid bilayer, and a comparative presentation of the proteins used in this study (*Hs*Δ29DHODH, *Ec*DHODH) with full-length *Hs*DHODH. The N-terminus of *Hs*DHODH contains a mitochondrial signal (MS) and a transmembrane segment (TM). These N-terminal parts of DHODH are connected to the catalytic domain via two alpha-helices (α1, α2) proposed to be critical for the interaction of *Hs*DHODH with the IMM, and the electron acceptor ubiquinone Q_10_ (Q). The proposed location of Q_10_ in the IMM is also shown, with the question mark referring to the following question addressed in our study: how does the enzyme interact with its co-substrate Q_10_? The numbering indicates the amino acid count of the full-length *Hs*DHODH sequence (Uniprot Q02127, PYRD_HUMAN), and indicates the shorter C-terminal length of *Ec*DHODH (Uniprot P0A7E1, PYRD_ECOLI). (**B**) Crystal structures for N-terminal truncated *Hs*DHODH (PDB ID: 2PRM) and *Ec*DHODH (PDB ID: 1F76). The α1-α2 microdomain is shown in red, the catalytic domain is shown in blue, a potentially flexible loop connecting the α1-α2 microdomain to the core catalytic domain with the co-factor FMN (depicted in orange) is shown in yellow.

*Hs*DHODH inhibitors also exhibit antiviral activity against a range of different viruses, with their effect being attributed to the depletion of the nucleosides that are necessary for the replication of the viral genome. *Hs*DHODH inhibition has therefore been proposed as a potential alternative intervention strategy in severe viral infections, e.g., caused by the Ebola virus [24,25] or respiratory RNA viruses, including the coronaviruses [26,27]. During the ongoing COVID-19 pandemic, several *Hs*DHODH inhibitors have been shown to inhibit the replication of SARS-CoV-2 (and other RNA viruses) in cell cultures [28,29,30]. It is therefore no wonder that approved anti-inflammatory *Hs*DHODH inhibitors, as well as compounds in clinical trials for AML, are now also tested for their effects in COVID-19 patients [28,31,32]. The bacterial *E. coli* DHODH (*Ec*DHODH) is peripherally associated with the cytosolic membrane [33] and uses ubiquinones as electron acceptors during aerobic growth, but must also be able to use alternative electron receptors (menaquinone, demethylmenaquinone) during anaerobic growth [34]. The structure and proposed orientations of *Hs*DHODH in the IMM and *Ec*DHODH at the cytosolic bacterial membrane are illustrated in Figure 1A, which also displays the proposed location of ubiquinone within the membrane. The N-terminus of *Hs*DHODH contains a bipartite signal consisting of a mitochondrial signal (MS) and transmembrane domain (TM) that determines its import and correct insertion into the IMM [13]. The N-terminal segments of Class II DHODHs are connected to the catalytic domain via a microdomain containing two alpha-helices (α1, α2). In the *E. coli* enzyme, this microdomain is an N-terminal patch of two alpha-helices followed by a short 3_10_-helix [9]. This microdomain is proposed to determine the interaction of DHODH with the membrane, and the binding of the electron acceptor ubiquinone. We will henceforth refer to this protein segment simply as the α1-α2 microdomain. In *Hs*DHODH and in the DHODH from *Rattus norvegicus*, this part of the enzyme is an important target for the binding of clinically used DHODH inhibitors, such as the active metabolite of ARAVA^®^ or atovaquone, and the former drug candidate brequinar [7,35,36]. In *Ec*DHODH, the structural elements corresponding to the MS and TM are missing, as illustrated in Figure 1A. 

Many crystal structures of the soluble catalytic domain of *Hs*DHODH in complex with inhibitors are currently available [7], but there are hardly any experimental structural data regarding the common feature of Class II DHODHs, the α1-α2 microdomain, or data concerning the enzyme in a membrane-bound state. To date, spectroscopic measurements indicate that the α1-α2 microdomain assumes a different conformation in detergent micelles and phospholipid vesicles [37], indicating that the nature of the membrane environment may play a role in the conformation adopted. Another interesting question is how the enzyme interacts with ubiquinone, which has been shown to be located in the hydrophobic center of lipid membranes [38], but it also needs to access the DHODH membrane-targeting α1-α2 microdomain as many DHODH inhibitors do [15]. Recently, it also has been proposed that inhibitor binding to DHODH is influenced by interactions with lipids in the IMM [39]. This is supported by previous findings showing differences in the inhibition of DHODH by brequinar, depending on whether the TM is present or not [35].

We therefore designed this study to understand the interaction of Class II DHODHs with membrane lipids and with the electron acceptor, ubiquinone. For our study, we chose the following two prominent members of Class II DHODHs: *Ec*DHODH and *Hs*DHODH. We performed a series of neutron reflectometry (NR) experiments in order to investigate the binding of both enzymes to a range of supported lipid bilayers with and without the co-substrate ubiquinone in order to determine what features of the membrane are important for the interaction with the enzymes and what role ubiquinone plays. These bilayers ranged from simple model bilayers that were prepared from synthetic lipids to complex lipid mixtures what were extracted from cell cultures. In order to focus on the proposed common feature of Class II DHODHs interaction with the membrane in this study, the α1-α2 microdomain, we compared the *Ec*DHODH with an N-terminal truncated version of *Hs*DHODH (*Hs*Δ29DHODH), which lacks the MS and TM domains (Figure 1A). *Hs*Δ29DHODH is comparable in enzymatic activity to the full-length *Hs*DHODH that is found naturally in mitochondria and the is most used variant of *Hs*DHODH studied throughout literature, also in interaction with lipids. Several reports of this, as well as comparisons of Class II DHODH with and without their N-terminal TM in vivo and *in vitro* studies have been published [13,35,40,41,42,43,44]. 

NR is a particularly well-suited technique for addressing these questions as it can determine the one-dimensional depth profile of single supported lipid membranes to 2–3 Å resolution, under solution conditions [45,46,47,48,49], and provide a low-resolution distribution profile of the embedded proteins using selective deuterium labeling of either the protein or the lipids. Recently developed methods allow the selective reconstitution of physiologically relevant membranes with a well-defined lipid composition [48]. NR is also well-suited for studying mechanisms and protein activity in situ [50,51,52], due to the flow-cell geometry of the samples. Additionally, the development of the synthesis of unsaturated deuterated lipids [53], and the purification of deuterated lipids from cell cultures [54], means that protein deuteration is not always required in order to achieve the required neutron contrast.

In mammalian cells, the inner mitochondrial membrane hosting *Hs*DHODH consists mainly of phosphatidylcholine (38–45%), phosphatidylethanolamine (32–39%), cardiolipin (14–23%), and phosphatidylinositol (2–7%) lipids. The outer mitochondrial membrane also consists mostly of phosphatidylcholine (44–59%) and phosphatidylethanolamine (20–35%) but contains a significantly lower amount of cardiolipin (1–10%) and a higher proportion of phosphatidylinositol (~13%) [55,56]. There are also variations in lipid composition depending on the tissue and species. In rats, for example, the cardiolipin content is significantly higher in the liver and kidneys (9–20%) than in the brain, lungs, or heart, where it ranges from 2 to 8% [55]. The *E. coli* cytosolic membrane that *Ec*DHODH [57] is associated with is composed of mainly phosphatidylethanolamine (76–77%), with phosphatidylglycerol and cardiolipin present at 11–12% each. Both the IMM and *E. coli* membranes are dominated by C16 and C18 acyl chain lengths, with the main differences being that *E. coli* membranes are more saturated and contain C17 cyclic fatty acids [58]. Cardiolipins, in particular, are mostly (~80%) composed of linoleic acyl chains in mammalian cells [59], whereas the acyl chains are primarily derived from palmitic acid in bacteria, such as *E. coli* [60]. 

As there are no published data on the interaction of DHODH with lipid membranes, we have focused on a selection of simple model membranes in order to begin to elucidate the relative membrane-binding strength of *Hs*Δ29DHODH and *Ec*DHODH and the dependence on the presence of ubiquinone and some of the lipids found in human mitochondria and the *E. coli* cytosolic membrane. Our results show that both of the enzymes bind to the membranes containing cardiolipin in the absence and presence of ubiquinone Q_10_, which is located at the center of the bilayers. *Ec*DHODH binds more efficiently to the surface of simple bilayers consisting of 1-palmitoyl, 2-oleoyl phosphatidylcholine (POPC), and tetraoleoyl cardiolipin (TOCL) compared to *Hs*Δ29DHODH. Q_10_ is located at the center of all of the lipid bilayers studied, including those prepared from POPC and TOCL, as well as those prepared from more complex lipid mixtures. We also show that both human and bacterial DHODH penetrate into the hydrophobic chain region of the outer lipid leaflet to meet the Q_10_, which remains in the central layer. We therefore show that the interaction between the enzymes and the membrane-embedded ubiquinone is mediated by enzyme penetration, and not by ubiquinone reorientation. The degree of enzyme penetration depends on the enzyme but also on the lipid composition of the bilayer. We hereby also highlight the importance of the lipid bilayer composition for the enzyme interaction. 

## 2. Results

### 2.1. General Experimental Setup

All of the experiments were executed following the same protocol. First, the supported lipid membranes were formed in situ in the NR sample cell by vesicle fusion after characterizing each Si-SiO_2_ surface by NR measurements in D_2_O and H_2_O. The lipid bilayer reflectivity was measured in four buffer contrasts consisting of different fractions of heavy water (D_2_O, CM4 (66 vol% D_2_O), CMSi (38 vol% D_2_O), and H_2_O). The protein solutions were prepared by diluting 5–10 mg/mL protein stock solutions in H_2_O buffer (in 20 mM Tris-HCl, 300 mM NaCl, 10 vol% glycerol, pH 7.4) to 0.4 mg/mL in 10 mM Tris-HCl, 100 mM NaCl, pH 7.4. For the D_2_O buffers, the pH was set to 7.0 in order to give a pD value of 7.4, since pD = pH + 0.4 at 25 °C. In D_2_O, this dilution scheme resulted in a minimum H_2_O content of 4–8 vol%. A total of 3 mL of protein solution was injected into the sample cell containing the lipid bilayer and the neutron reflectivity was measured after 30 min of incubation in the same solvent contrast, as well as after the sample cell was rinsed with buffer. Protein addition and rinsing were repeated in every contrast due to the protein interaction being partially reversible, as shown by our previous QCM-D measurements [43]. An overview of all of the bilayers and enzymes measured can be found in Supplementary Material Table S1. Previously published component volumes of the lipid constituents [61,62,63,64,65] were used to calculate the scattering length density (SLD) values shown in Table S2. Previously published amino acid molecular volumes [66] were used to calculate the SLD values for the proteins under study (Table S2) taking into account the exchange of protons with the solvent in each buffer contrast.

In all of the systems studied, addition of protein resulted in the formation of multiple protein layers (two to six) on top of the lipid bilayer. It should be noted, however, that these additional layers are very sparsely populated and consist mostly of water, and they are thus unlikely to influence the behavior of the inner protein layer. As we use D_2_O for contrast, we wanted to exclude the isotope effects on protein stability and aggregation and therefore tested the influence of the different contrasts (containing different amounts of D_2_O) on protein stability and aggregation. For this, we performed thermal stability experiments with nano differential scanning fluorimetry (nanoDSF) in different buffer contrasts. We could not find differences in the T_m_ values of the proteins in the different contrasts, or any signs of induced protein aggregation (Table S3). It is, however, possible that the formation of multiple protein layers is related to some nonspecific protein–protein interactions becoming evident due to the length of the experiments or the local protein concentration at the bilayer surface, but as it is unlikely to be physiologically relevant, we have mainly focused on the details of the first protein layer in contact with the lipid membranes and the degree of protein penetration into the lipid bilayer. For this reason, the terms “retention” and “binding strength” in this manuscript refer to the first protein layer that is in contact with the lipids. By binding strength, we refer to the observed amount of bound enzyme in the first protein layer, and by retention, we do not refer to the equilibrium dissociation constant, but rather to whether or not the bound protein is displaced by rinsing with buffer.

### 2.2. EcDHODH Binds the Surface of POPC/TOCL Membranes More Strongly Than HsΔ29DHODH, but Penetrates into the Bilayer Less Efficiently 

Our previous results, which were obtained with quartz crystal microbalance with dissipation monitoring (QCM-D) studies [43], showed that both *Hs*Δ29DHODH and *Ec*DHODH have very low binding to pure POPC lipid bilayers and bind reversibly, and that TOCL is a prerequisite for more stable binding. We therefore decided to investigate the structural basis of their interaction with the bilayers containing POPC and 10 mol% TOCL using NR. In the inner mitochondrial membrane of mammalian cells, the fraction of cardiolipin ranges from 10 to 20 mol% [55]. In order to ensure enough neutron contrasts to determine the structure of the protein-containing bilayers, we measured the binding of *Hs*Δ29DHODH to the bilayers that were prepared with either hydrogenous POPC or chain-deuterated POPC (d_63_-POPC) [67] in combination with hydrogenous cardiolipin (TOCL). Measuring these bilayers in four different buffer solution contrasts before and after protein addition and after rinsing allowed us to determine the lipid composition (amount of TOCL and Q_10_) and solvent fraction in each layer in addition to the lipid and protein layer thicknesses. The analysis procedure is described in the Materials and Methods section. We also investigated the binding of *Ec*DHODH to a bilayer that was prepared from POPC and TOCL.

**Figure 2:**
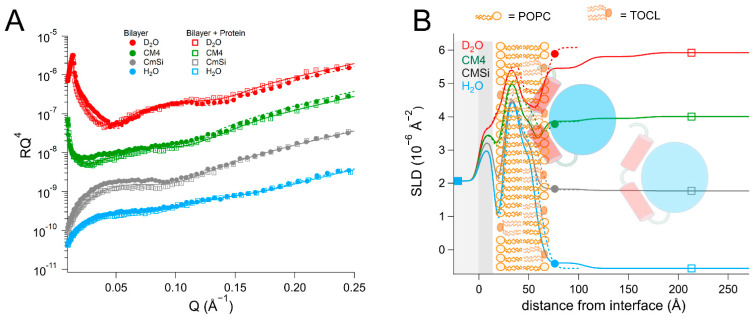
(**A**) Reflectivity curves (data from INTER, ISIS) and (**B**) SLD profiles for d_63_-POPC/TOCL membranes before and after addition of *Hs*Δ29DHODH, with a schematic representation of the model structure. POPC molecules are shown in brown (hollow heads, two tails). TOCL molecules are depicted in orange (filled heads, four tails). The α1-α2 microdomain of the protein is shown in red and the catalytic domain is depicted in blue.

The NR data from the bilayers consisting of POPC and TOCL were modeled using the following four laterally homogenous layers: inner lipid headgroups, inner lipid chains, outer lipid chains, and outer lipid headgroups. For the bilayer that were prepared with 90 mol% d_63_-POPC and 10 mol% TOCL (Table 1 and Table S4, and Figure 2), the selective deuteration allowed us to observe an asymmetric distribution of the two lipid species between the leaflets. A total of 10 mol% TOCL corresponds to 20.5 vol% of the total lipid chain volume, but the data could be best fitted by a model in which the inner membrane leaflet contains 14 ± 2 vol% TOCL (SLD = 5.4 ± 0.1 × 10^−6^ Å^−2^) and the outer leaflet 27 ± 2 vol% TOCL (SLD = 4.6 ± 0.1 × 10^−6^ Å^−2^), corresponding to a total TOCL content of 21 ± 2 vol%. This spontaneous asymmetry arises from the cardiolipin headgroup being negatively charged, making its interaction with the negatively-charged SiO_2_ surface less favorable. The bilayer had a surface coverage (total lipid volume fraction in the chain region) of 81 ± 3 vol%, indicating that the surface had 19 ± 3 vol% bilayer-free area containing solvent. The data from the bilayers that were prepared using hydrogenous lipids (90 mol% POPC and 10 mol% TOCL) could be fitted using a similar structure (Table S5 and Figure S1) with a surface coverage of 91 ± 2 vol%. 

The addition of *Hs*Δ29DHODH to the d_63_-POPC/TOCL bilayer resulted in the formation of two very hydrated protein layers on top of the lipid bilayer of 43 ± 5 Å and 60 ± 15 Å thickness. The re-addition of protein solutions after each buffer rinse in each of the contrasts gave rise to small variations in the solvent content and the thickness of the protein layers, but most of the protein remained bound. The repeated protein addition and rinsing did not result in significant changes in the lipid bilayer coverage. Most notably, the protein binding resulted in a clear decrease in the SLD value of the outer lipid chain layer, but no change in the inner lipid layer. This indicates that some of the protein penetrates into the lipid chains, as the values cannot be explained by the redistribution of the lipids. The SLD of the outer chain layer corresponds to 37 ± 8 vol% protein, relative to the lipid chains, and makes up 29 ± 8 vol% of the whole layer, taking into account the solvent fraction (21 ± 3 vol%). Protein SLD values vary with solvent contrast due to proton exchange with the solvent, and in this case, although the solvent variation is close the fitting uncertainty in the lipid chain region, including it does lead to an improvement in the quality of the fits. However, some of the SLD change can also arise from a disturbed lipid packing due to the protein penetration. In the outer headgroups that contain 44 ± 5 vol% solvent, the resolution to the protein is poorer but the data are consistent with the same absolute protein volume fraction as in the chains (29 ± 8 vol%). The binding of *Hs*Δ29DHODH to the hydrogenous (POPC/TOCL) bilayers was measured in two solvent contrasts (Table S5 and Figure S1) and could also be modeled using a model of two adsorbed protein layers containing 93 ± 3 and 98 ± 3 vol% solvent, but without explicitly including protein penetration due to the poorer contrast.

The dimensions of *Hs*Δ29DHODH, according to the crystal structure corresponding to the ligand-free enzyme (PDB ID: 2PRM) [15], are approximately 45 Å on the vertical axis (with the alpha-helical domain pointing downwards) and 53 Å on the horizontal axis (Figure 1B). The approximate volume of a single protein molecule based on the amino acid composition (Table S2) is 49165 Å^3^. The addition of *Hs*Δ29DHODH on the d_63_-POPC/TOCL bilayer results in the adsorption of 2.8 ± 1.1 × 10^5^ molecules/µm^2^ and a protein–lipid ratio of 1:44 in the outer leaflet (based on the most sensitive contrast). 

**Figure 3:**
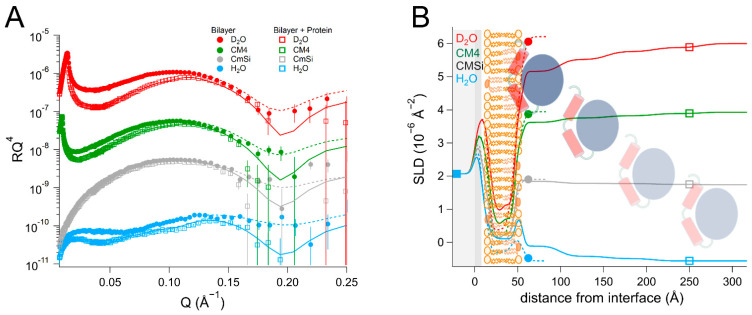
(**A**) Reflectivity curves (data from D17, ILL) and (**B**) SLD profiles for POPC/TOCL membranes before and after addition of *Ec*DHODH, with a schematic representation of the model structure. POPC molecules are shown in brown (hollow heads, two tails). TOCL molecules are depicted in orange (filled heads, four tails). The α1-α2 microdomain of the protein is shown in red and the catalytic domain is depicted in blue.

The addition of the bacterial enzyme (*Ec*DHODH) to a POPC/TOCL bilayer (Table 2 and Table S6, and Figure 3) also resulted in a change in the SLD of the lipid chain region that could not be explained only by the presence of solvent or lipid rearrangement. Some of the protein initially occupied the defects in the lipid bilayer and displaced the solvent in the H_2_O contrast, but in all of the subsequent contrasts, the lipid–protein interaction could be modelled in a manner similar to that of the human enzyme, assuming that the protein binds as several layers on top of the lipid bilayer and penetrates into the outer lipid chains. The first protein layer is more densely populated than in the case of the human enzyme, displaying a solvent content of 72 ± 2 vol%, but the penetration into the outer lipid chain layer is weaker, where it accounts only for 8 ± 3 vol% of the lipid chain volume. This suggests that there is a stronger interaction of the bacterial protein with the lipid bilayer surface compared to *Hs*Δ29DHODH, but a weaker interaction with the lipid chains. However, an increased solvent content of 19 ± 2 vol% was also observed in the lipid chains, indicating that the protein removes some of the lipids (9 vol%) upon rinsing, and that some of the protein may be removed along with them. However, the protein–lipid interaction is less reversible than for the truncated human enzyme, as the enzyme and lipid amounts remain stable between subsequent contrasts/rinsing. The innermost protein layer has a thickness of 46 ± 5 Å, while the subsequent layers can be modeled with a thickness of 55 ± 15 Å each and an increasing solvent content.

### 2.3. DHODH Penetration into the Lipid Hydrophobic Region Mediates Interaction with Q_10_ Located at the Center of POPC/TOCL Bilayers

We proceeded to investigate the location and the effect of Q_10_ on the binding of DHODH in POPC and TOCL bilayers. In order to ensure enough contrast to detect the location of the ubiquinone, we measured the binding of *Hs*Δ29DHODH to bilayers prepared with either POPC or d_63_-POPC in combination with TOCL and Q_10_. We then compared the binding of *Ec*DHODH to *Hs*Δ29DHODH to a bilayer that was prepared from POPC, TOCL, and Q_10_. 

**Figure 4:**
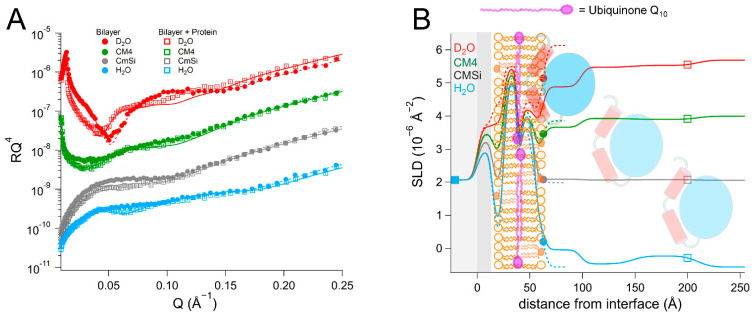
(**A**) Reflectivity curves (data from INTER, ISIS) and (**B**) SLD profiles for d_63_-POPC/TOCL/Q_10_ membranes before and after addition of *Hs*Δ29DHODH. POPC molecules are shown in brown (hollow heads, two tails). TOCL molecules are depicted in orange (filled heads, four tails). The α1-α2 microdomain of the protein is shown in red and the catalytic domain is depicted in blue. Ubiquinone molecules are represented in purple (filled heads, long tails).

The d_63_-POPC/TOCL/Q_10_ bilayer could not be modelled using a four-layer model, as was the case for the d_63_-POPC/TOCL bilayers. It was therefore modelled using the following five layers (Table 3 and Table S7, and Figure 4): inner lipid heads, inner lipid chains, ubiquinone + lipid chains, outer lipid chains, and outer lipid heads. d_63_-POPC and TOCL could be assumed to be asymmetrically distributed between the inner and outer leaflets, as observed in the case of the d_63_-POPC/TOCL bilayers. The inner lipid chains displayed an SLD of 5.4 ± 0.2 × 10^−6^ Å^−2^ (corresponding to 86 ± 3 vol% POPC and 14 ± 3 vol% TOCL) and the outer lipids chains showed an SLD of 4.4 ± 0.2 × 10^−6^ Å^−2^ (71 ± 3 vol% POPC and 29 ± 3 vol% TOCL), which are in very good agreement with the SLD values corresponding to the d_63_-POPC/TOCL bilayers. Ubiquinone was found to be concentrated in a separate layer in the middle of the bilayer, which was 4 ± 1 Å thick and consisted of 51 ± 5 vol% Q_10_ and 49 ± 5 vol% phospholipid chains based on the SLD value corresponding to the best fit (2.7 ± 0.2 × 10^−6^ Å^−2^) in the most sensitive contrast. The total amount of ubiquinone in this layer correlates well with the 10 mol% added to the lipids. The inner and outer lipid chain layer thickness values were also smaller than in the absence of Q_10_, which is consistent with part of the lipid chains being located in the middle ubiquinone-rich layer. The solvent content in the entire lipid chain region was 9 ± 1 vol%, corresponding to a 91 ± 1 vol% bilayer coverage, whereas the total bilayer thickness was 49 ± 1 Å. A ubiquinone-rich middle layer was also observed in the POPC/TOCL/Q_10_ bilayers (Table S8 and Figure S2), in which Q_10_ accounted for 51 ± 13 vol%, with the uncertainty being larger due to the lower contrast between Q_10_ and the non-deuterated phospholipid chains. The bilayer coverage was 95 ± 1 vol% and the thickness was 49 ± 1 Å. In summary, these results indicate clearly that Q_10_ localizes to the center of the bilayer. 

The addition of *Hs*Δ29DHODH to the d_63_-POPC/TOCL/Q_10_ bilayer resulted in the formation of three protein layers on top of the lipids, the first layer had a thickness of 36 ± 5 Å and 70 ± 2 vol% solvent in the most sensitive contrast (D_2_O). In the first contrast measured (H_2_O), the protein appeared to penetrate into the solvent-filled defects in the bilayer, as the solvent fraction decreased to 0 ± 2 vol%, while the inner and outer chain SLD values decreased to 5.0 ± 0.2 × 10^−6^ Å^−2^ and 4.0 ± 0.2 × 10^−6^ Å^−2^, respectively, which corresponds to 11 vol% of the bilayer being composed of protein. In subsequent contrasts, the protein addition resulted in a decrease in the SLD of the outer lipid chain layer (from 4.4 ± 0.2 × 10^−6^ Å^−2^ to 4.0 ± 0.2 × 10^−6^ Å^−2^ in D_2_O) and an SLD variation with contrast that is consistent with 29 ± 14 vol% DHODH relative to the lipids. The best fit suggests, however, that the SLD of the middle ubiquinone layer remains unchanged (2.7 ± 0.2 × 10^−6^ Å^−2^). These SLD changes suggest that the protein penetrates the outer lipid chain region but that no ubiquinone reorientation or migration occurs towards the lipid–water interface from the middle layer. The protein binding does not seem to remove a significant portion of the lipid bilayer, as the bilayer coverage remains stable at 89 ± 2 vol%. As in the ubiquinone-free d_63_-POPC/TOCL bilayer, protein binding could also be modeled in the outer lipid headgroups with the same absolute volume fraction as found in the lipid chain layer. 

The results obtained with the fully hydrogenous bilayers (POPC/TOCL/Q_10_) upon *Hs*Δ29DHODH binding (Table S8 and Figure S2) are also consistent with uniform protein penetration into the outer lipid chain region. The SLD of the lipid chain region increases from −0.27 ± 0.1 × 10^−6^ Å^−2^ to −0.17 ± 0.1 × 10^−6^ Å^−2^, while the SLD of the inner lipid region remains unchanged. This suggests that there is only a small amount of protein penetration into the outer lipid chain region, 3 ± 3 vol% relative to the lipids. There is no evidence of ubiquinone migration, as the SLD of the ubiquinone middle layer remains unchanged. The protein addition also results in the formation of three protein layers on top of the lipid bilayer. The innermost protein layer is 38 ± 5 Å thick and contains 78 ± 2 vol% water in the most sensitive contrast (D_2_O). The results obtained with the hydrogenous bilayers also confirmed that the lipid chain region of the bilayer did not become significantly thicker or thinner as a result of protein addition. 

Rinsing with the buffer resulted in the removal of 33 ± 10% of the protein that was initially bound to the d_63_-POPC/TOCL/Q_10_ bilayer and the solvent content of the innermost protein layer increased to 80 ± 2 vol% in the most sensitive contrast. Rinsing removed 45 ± 16% of the protein initially bound to the POPC/TOCL/Q_10_ bilayer. Rinsing did not result in additional changes in the SLD values of the ubiquinone and outer lipid chain regions for any of the datasets studied. 

**Figure 5:**
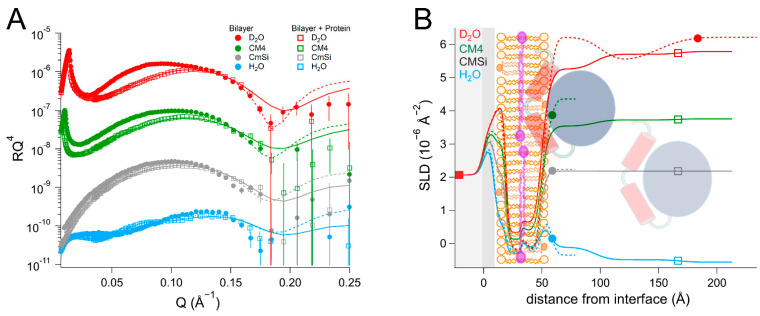
(**A**) Reflectivity curves (data from INTER, ISIS) and (**B**) SLD profile for POPC/TOCL/Q_10_ bilayers before and after addition of *Ec*DHODH. POPC molecules are shown in brown (hollow heads, two tails). TOCL molecules are depicted in orange (filled heads, four tails). The α1-α2 microdomain of the protein is shown in red and the catalytic domain is depicted in blue. Ubiquinone molecules are represented in purple (filled heads, long tails).

In the POPC/TOCL/Q_10_/*Ec*DHODH system (Table 4 and Table S9, and Figure 5), protein addition resulted in the formation of two layers on top of the lipid bilayer, the first with a thickness of 39 ± 5 Å, and the outer layer had a thickness of 73 ± 15 Å. The solvent in the protein inner layer amounted to 81 ± 2 vol% in all of the contrasts, suggesting a stable protein–lipid interaction. As with the human enzyme, the SLD of the ubiquinone middle layer remained unchanged but the SLD of the outer lipid chain region increased to 0.057 ± 0.2 × 10^−6^ Å^−2^ in the D_2_O contrast, with an SLD variation with contrast indicating that 10 ± 8 vol% relative to the lipids of protein penetrates into the chains. The protein binding also resulted in some of the lipids being removed, as indicated by an 8 ± 2 vol% decrease in the bilayer coverage. Rinsing removed very little of the initially bound protein and the solvent content remained almost unchanged (85 ± 2 vol% in H_2_O). No major changes were observed in the SLD values of the bilayer after rinsing. 

Compared to the binding of *Ec*DHODH to membranes consisting only of POPC and TOCL (Table 2), the incorporation of Q_10_ slightly increased the amount of the enzyme penetrating into the lipid bilayer, while decreasing the overall amount of enzyme on the bilayer surface, as only two protein layers were observed in the presence of Q_10._ The protein density in the innermost layer was similar.

### 2.4. The Presence of Q_10_ Does Not Increase Protein Binding Strength or Retention in Complex Lipid Bilayers

**Figure 6:**
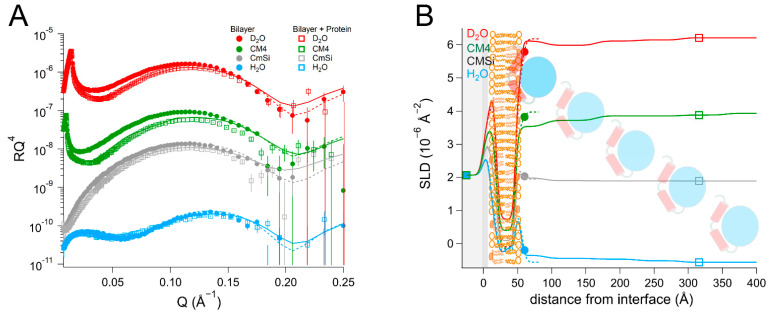
(**A**) Reflectivity curves (data from D17, ILL) and (**B**) SLD profiles for yeast membranes before and after addition of *Hs*Δ29DHODH. The α1-α2 microdomain of the protein is shown in red and the catalytic domain is depicted in blue. The complex mixture of the yeast membranes is schematically depicted here using the same image as for POPC/TOCL in previous figures.

**Figure 7:**
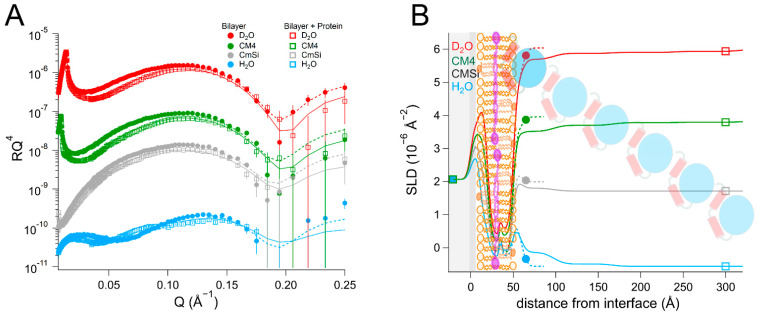
(**A**) Reflectivity curves (data from D17, ILL) and (**B**) SLD profiles for yeast lipid bilayers supplemented with Q_10_ before and after addition of *Hs*Δ29DHODH. The α1-α2 microdomain of the protein is shown in red and the catalytic domain is depicted in blue. Ubiquinone molecules are represented in purple (filled heads, long tails). The complex mixture of the yeast membranes is schematically depicted here using the same image as for POPC/TOCL in previous figures.

We then proceeded to investigate the interaction of *Hs*Δ29DHODH with bilayers of increased lipid complexity. For this we used a phospholipid mixture extracted as described in [48] from the yeast *Candida glabrata* [68], from which we prepared membranes in the presence and absence of 10 mol% Q_10_. The phospholipid composition of this mixture was (mol%) as follows: 52% phosphatidylcholine (PC), 27% phosphatidylserine (PS), 14% phosphatidylethanolamine (PE), 4% phosphatidylinositol (PI), and 3% cardiolipin (CL). The fatty acid distribution (mol%) was as follows: 40% C18:1, 38% C16:1, 11% C18:0, 6% C16:0, 3% C16:2, 4% C18:2, and 2% C18:3, as reported in [69]. For comparison, the molar lipid composition of the inner mitochondrial membrane of mammalian cells is approximately 45% PC, 30% PE, 20% CL, and the rest corresponds to other lipids (mostly PI) [55]. The average headgroup lipid volume of the yeast lipid mixture calculated from the composition is 305 Å^3^ and that of the tails is 942 Å^3^. The data from this bilayer could be fitted using the expected SLD values for both the lipid chains and the headgroups (Table 5 and Table S10, and Figure 6). The theoretical SLD values for the lipid chains and headgroups are listed in Table S2. In the fits, the different lipid classes were assumed to be symmetrically distributed between the inner and outer leaflets to within the sensitivity to the lipid composition, in the absence of selective deuteration to detect asymmetry. The bilayers that were prepared with Q_10_ could be modelled using a similar model of five layers (inner heads, inner chains, middle layer, outer chains, and outer heads), as for POPC/TOCL bilayers. The middle ubiquinone rich layer was also found to be of the same thickness, 4 ± 1 Å, and composed of 57 ± 14 vol% Q_10_ (Table 6, Table S11, and Figure 7).

The addition of *Hs*Δ29DHODH to the yeast phospholipid bilayer resulted in the formation of several increasingly hydrated protein layers on top of the bilayer. The innermost layer had a thickness of 35 ± 5 Å and solvent content of up to 70 ± 2 vol%. The outer protein layers were 60 ± 15 Å to 75 ± 15 Å thick. As was the case for the synthetic lipid bilayers, the protein penetrated through the outer headgroups and into the outer lipid chains. The protein accounted for 10 ± 5 vol% of the volume in the mixed lipid chain/protein layer. The protein addition also resulted in some lipid removal, as indicated by the solvent content in the lipid chain region, which increased from 2 ± 1 vol% to 15 ± 1 vol%. In the case of the bilayer containing Q_10_, the protein resulted in very similar layers and penetration profile into the lipid bilayer as without Q_10_ (Table 5 and Table S10, and Figure 6). 

The rinsing removed a large fraction of the protein that was initially bound to bilayers both with and without ubiquinone. These results also suggest that, in contrast to what we observed for the bilayers that were prepared with synthetic lipids, ubiquinone does not increase the binding of *Hs*Δ29DHODH to more complex bilayers containing other lipids. Compared to the bilayers consisting of synthetic lipids (POPC, TOCL), the bilayers that were prepared with lipid mixtures extracted from yeast resulted in a higher degree of *Hs*Δ29DHODH binding. In other words, the lipid composition does have a major effect on protein binding strength.

**Figure 8:**
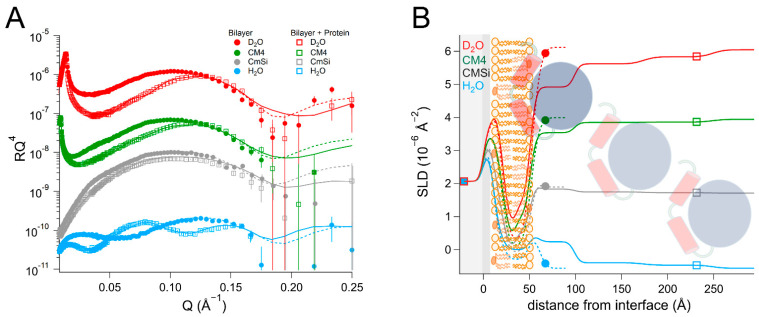
(**A**) Reflectivity curves (data from D17, ILL) and (**B**) SLD profiles for bacterial mimic membranes before and after addition of *Ec*DHODH. The α1-α2 microdomain of the protein is shown in red and the catalytic domain is depicted in blue. The mimic mixture of the bacterial membrane is schematically depicted here using the same image as for POPC/TOCL in previous figures.

Finally, we investigated the interaction between the DHODH from *E. coli* and a mixture of synthetic lipids mimicking the composition of the bacterial plasma membrane [70]. The composition (mol%) of the lipid mixture used was as follows: 40% POPC, 35% POPE, 13% POPG, and 12% TOCL. In this case, the data from the resulting bilayer (Table 7 and Table S12, and Figure 8) could be fitted with the expected SLD values, the bilayer coverage was 91 ± 1 vol%, and the total thickness was 50 ± 1 Å.

After protein addition, *Ec*DHODH was observed to form three layers of increasing hydration on top of the lipid bilayer. The inner protein layer had a thickness of 40 ± 5 Å and a solvent content of 63 ± 2 vol% in the final contrast measured (D_2_O). The outer protein layers were between 55 ± 5 Å and 60 ± 5 Å thick and had higher levels of hydration. The protein penetrated into the outer lipid chains and accounted for 25 ± 6 vol% relative to the lipids. This is considerably more than the amount of *Ec*DHODH penetrating into the POPC/TOCL and POPC/TOCL/Q_10_ and indicates that the other lipids native to *E. coli* (POPE and POPG) are important for binding. The rinsing removed no significant amount of the initially bound protein. 

In summary, the only common model describing all of the experimental data sets features the homogeneous penetration of *Hs*Δ29DHODH and *Ec*DHODH into the outer lipid leaflet. The experiments presented above suggest that both the presence of the substrate Q_10_ and the composition of the lipid bilayer play a determining role for the relative binding strength of the enzyme to the bilayer. 

## 3. Discussion

To the best of our knowledge, our study is the first in situ structural investigation of the interaction between lipid bilayers and Class II DHODHs, in which both the lipid and the protein structures are resolved in one dimension. This provides the benefit of observing how the lipid bilayer structures differ based on their composition and how this influences the interaction with the protein. 

To date, the interaction of *E. coli* DHODH with mixed DOPC/Triton X-100 vesicles has been investigated by electronic spin resonance [71] and the ability of N-terminally Class II truncated *Plasmodium falciparum* DHODH to bind to PC and PE liposomes was shown by size-exclusion chromatography [72]. Furthermore, the α1-α2 microdomain in *Hs*DHODH, as an isolated synthetic peptide, has also been studied. Using this peptide, spectroscopic measurements indicate that the α1-α2 microdomain assumes a different conformation in detergent micelles and phospholipid vesicles [37].

These studies indicate that DHODHs lacking transmembrane domains, such as the one from *E. coli*, can interact with lipid bilayers and that the α1-α2 microdomain undergoes conformational changes depending on the interaction partner. Therefore, it is likely that the lipid composition plays a major role on the protein–membrane interactions. In this study we set out to investigate this phenomenon. A second question that we addressed is how DHODHs belonging to Class II might interact with the co-substrate, ubiquinone, and how this affects protein–membrane interactions.

By using multidimensional neutron contrast variation of the lipids and the aqueous solvent, we have directly observed the location of ubiquinone in the bilayers, consistent with previous experiments with neutron diffraction [38], showing that ubiquinone tends to localize at the center of the lipid bilayer in multilamellar lipid stacks and fills the interstitial space in the inverse hexagonal H_II_ phase of POPE [73]. Our results clearly indicate a perpendicular orientation of Q_10_ relative to the phospholipids. We could show this location in a variety of bilayers, including those that were prepared from complex lipid mixtures derived directly from a eukaryotic organism (*Candida glabrata*), translating previous findings with two synthetic lipids alone [38] to a more complex and physiologically relevant setting. Our most significant finding is that the binding of both *Hs*Δ29DHODH and *Ec*DHODH to the surface of the lipid bilayers does not result in the migration or reorientation of ubiquinone from the middle layer towards the membrane–water interface where the protein is located. Instead, our data show that, upon binding to the lipid bilayer, both of the enzymes penetrate into the outer lipid chain region, both in the absence and presence of Q_10_. A direct interaction between Q_10_ and the enzyme is suggested by the increased retention upon rinsing that *Hs*Δ29DHODH displays on POPC/TOCL bilayers containing Q_10_ in comparison to those without. This effect was both observed in our study here and in our previous study [43] using QCM-D. The location of ubiquinone at the center of the lipid bilayers and the lack of observable reorientation in the bilayer to bind DHODH are consistent with its large molecular size and branched chain structure, which make it poorly soluble in the phospholipids. 

In most cases, the inner protein layer on the surface of the lipid bilayer is somewhat thinner (35–46 Å) than the protein dimensions in the crystal structure and suggest the formation of a monolayer of the protein. However, according to the data analysis, the proteins also penetrate into the outer lipid leaflet by up to 23–24 Å, which would make the total thickness of the first protein layer clearly greater than the crystal structure suggests. Two possible scenarios could explain this. The crystallographic data indicates the α1-α2 microdomains of both of the enzymes (Figure 1B) are connected to their respective core catalytic domains by potentially flexible loops. Movements in this loop have been shown upon inhibitor binding to N-terminal truncated *Hs*DHODH and reveal the possibility of conformational flexibility in this region of DHODHs [15]. Although there are no published reports of this to date, it is likely that the amphipathic α1-α2 microdomain penetrates into the outer lipid chain region, whereas the rest of the catalytic domain remains on the surface of the bilayer, such a conformational rearrangement is facilitated by the flexible loop, as mentioned previously. Alternatively, it is possible that two partially overlapping layers of protein are in contact with the lipid bilayer, one penetrating into the lipids and one on the surface. Our NR data is consistent with both of these scenarios, however it could also be explained by a combination of α1-α2 microdomain penetration and a second, partially overlapping protein layer on the membrane surface. The higher thicknesses of the outer protein layers are less defined, due to the very high solvent fraction (>90 vol% water) and do not necessarily portray individual protein layers.

**Figure 9:**
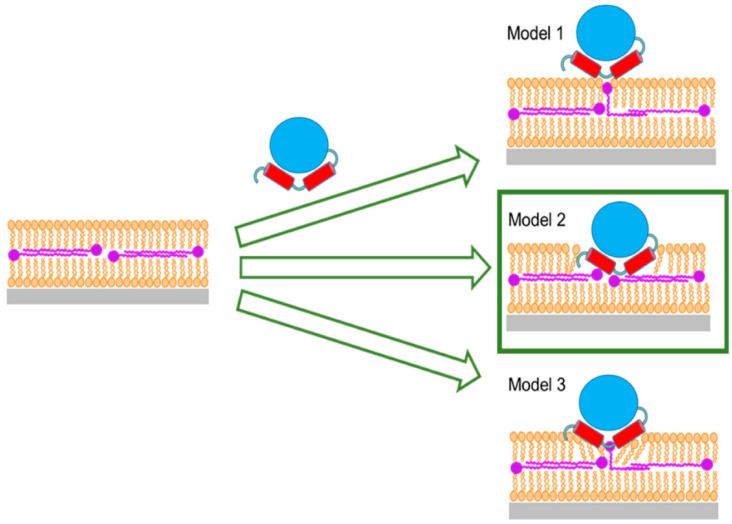
Suggested models for the interaction of Class II DHODHs with ubiquinone. Model 1: Q_10_ bends up and reaches toward the enzyme at the outside of the lipid bilayer. Model 2: The enzyme penetrates the whole outer lipid bilayer leaflet to reach the Q_10_ at the center of the bilayer. Model 3: The enzyme partially penetrates the outer lipid bilayer leaflet and Q_10_ bends up and reaches towards the enzyme. Model 2, marked by a green box, is the only scenario supported by our data.

The observation that both *Hs*Δ29DHODH and *Ec*DHODH clearly penetrate into the lipid bilayer to a significant extent is new, to our best knowledge, and indicates that the interaction of the α1-α2 microdomain with lipids has perhaps a larger role to play in the membrane–DHODH interaction than previously thought. This is particularly interesting in the context of the present comparison of the naturally soluble *Ec*DHODH for which this is the main interaction with membrane lipids, with the truncated *Hs*Δ29DHODH, which in vivo has a transmembrane domain anchoring it to the IMM. The differences that were observed in the membrane-binding strength and reversibility of the lipid interaction for the two enzymes support a stronger ability of *Ec*DHODH to bind to the bacterial plasma membrane using only the alpha-helical domain, whereas *Hs*Δ29DHODH also requires the TM to remain in the IMM. The model of enzyme penetration towards the ubiquinone located at the center of lipid bilayers (Figure 9) could be relevant for other enzymes that use ubiquinones as electron acceptors. Ubiquinones are found in all membranes, but Q_10_ is the key node in the mitochondrial respiratory chain and a substrate for other enzymes comparable to DHODH, such as succinate dehydrogenase, glycerol-3-phosphate dehydrogenase, or electron transfer flavoprotein coenzyme Q, located at the IMM [74,75]. In summary, our results provide evidence suggesting that the protein–ubiquinone interaction is facilitated by penetration of the enzyme into the outer lipid chain region (Model 2 in Figure 9), and, not to any degree observable in our experiments using NR, by migration of ubiquinone from the middle layer towards the outer lipid chain with enzyme partially, or not at all penetrating. 

The presence of ubiquinone increases the binding of *Hs*Δ29DHODH to bilayers consisting of synthetic lipids (POPC, TOCL), as indicated by a more densely populated innermost protein layer (containing less solvent), which is also more stable, as indicated by a lower degree of protein removal by rinsing. Lipid composition also has an effect on the binding of *Hs*Δ29DHODH to the lipid bilayers. The bilayers consisting of a eukaryotic phospholipid mixture that were derived from yeast display a significantly higher protein binding ability compared to the bilayers that were prepared from synthetic lipids. However, incorporation of Q_10_ into bilayers prepared with this complex mixture of lipids does not result in a significantly increased binding of the enzyme or a higher retention, as is the case with synthetic lipid bilayers. Our results are consistent with previous studies using non-denaturing electrospray ionization mass spectrometry (nESI-MS) that have inferred that an N-terminally truncated *Hs*DHODH, resembling our *Hs*Δ29DHODH, displays a higher relative binding to lipids, such as TOCL and POPE, compared to POPC [40], as they are detected by MS in protein–lipid complexes. 

We previously demonstrated, by using QCM-D experiments, that both *Hs*Δ29DHODH and *Ec*DHODH bind much more strongly to POPC bilayers that contain 10 mol% of TOCL, whereas in its absence the binding of both of the enzymes is reversible [43]. As the lipid chains are similar in both POPC and TOCL, this suggests that the cardiolipin headgroup is predominantly responsible for this effect. We therefore focused, in this study, on the effect of the lipid headgroups and the electrostatic interaction with the enzyme, by matching the acyl chains of the CL, PE, and PG to the POPC-based samples. The composition of the yeast lipid bilayer that was used was 52 mol% phosphatidylcholine (PC), 27 mol% phosphatidylserine (PS), 14 mol% phosphatidylethanolamine (PE), 4 mol% phosphatidylinositol (PI), and 3 mol% cardiolipin (CL) [69], and the fatty acid composition of the mixture was as follows (mol%): 40% oleic, 38% palmitoleic, 11% stearic, 6% palmitic, 4% linoleic, and 2% linolenic [69]. The CL in *C. glabrata* has the native acyl chain distribution similar to the overall mixture, apart from the increased linoleic acid (37.4% oleic, 33.7% palmitoleic, 11.3% linoleic, 9.1% palmitic, and 8.6% stearic). Compared to the 90 mol% POPC/10 mol% TOCL and the 80 mol% POPC/10 mol% TOCL/10 mol% Q_10_ bilayers, the yeast lipid mixture contains fewer neutral lipids (POPC) and more negatively-charged lipids, such as PS and CL. In line with what has been suggested by Costeira-Paulo et al. [40], we hypothesize that the electrostatic interactions between these negatively-charged lipid headgroups and the cationic residues present in the amphipathic α1-α2 microdomain of *Hs*Δ29DHODH are likely to be the main drivers for protein–membrane interaction. However, as there are also differences in the lipid chain composition of the cardiolipins found in human mitochondria and in *E. coli*, with the latter being more saturated, an additional role for the lipid chains in the binding that was observed here for the two truncated enzymes can certainly not be excluded and deserves to be investigated in future studies.

Our results indicate that the bacterial *Ec*DHODH displays a higher degree of binding to POPC and TOCL compared to the truncated human enzyme (*Hs*Δ29DHODH). The interaction between the bacterial enzyme and the lipids is both stronger and more stable. The presence of ubiquinone in the lipid bilayer does not significantly increase the binding of *Ec*DHODH. The lipid complexity does have an effect on *Ec*DHODH binding, as its binding to bilayers, mimicking the composition of the bacterial plasma membrane (i.e., containing POPE and POPG), is even higher compared to only POPC and TOCL. 

**Figure 10:**
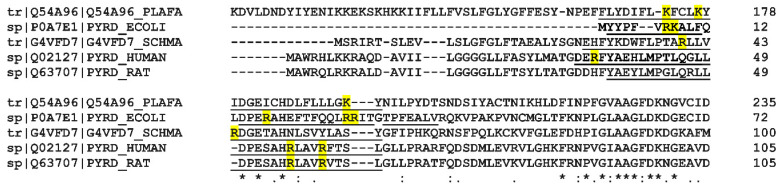
Partial multiple sequence alignment of the N-terminal region of Class II DHODHs, of which a crystal structure including the α1-α2 microdomain is available (the full alignment can be found in the Supplementary Material Figure S3). The alignment was performed with CLUSTAL OMEGA(1.2.4) [77]. PLAFA: *Plasmodium falciparum* PDB 6I55; ECOLI: *Escherichia coli* PDB 1F76; SCHMA *Schistosoma mansoni* PDB 6UY4; HUMAN: *Homo sapiens* PDB 2PRM; RAT: *Rattus rattus* PDB 1UUM. The respective UniProt identifiers for the amino acid sequences used in the alignment are given to the left of each row between vertical lines. Amino acid stretches corresponding to α1-α2 microdomain according to the PDB entries are underlined. Cationic amino acid residues in these regions are marked in yellow.

In order to examine the structural features of Class II DHOHDs, we searched the UniProt Database (accessed 16 December 2020) for Class II DHODHs with available X-ray crystal structures using the following query search in PDB: “dihydroorotate dehydrogenase quinone database: (type:pdb)”. From the returned eight hits, three (the DHODHs from *Eimeria tenella*, *Helicobacter pylori,* and *Mycobacterium tuberculosis*) had obsolete (not available) PDB entries, or did not cover the full α1-α2 microdomain. The remaining five sequences were used to perform a multiple sequence alignment (Figure 10 and Figure S3).

We hypothesize that the higher relative binding displayed by the bacterial enzyme may arise from the presence of an abundance of positively charged residues on the outer surface of the α1-α2 microdomain of *Ec*DHODH (Arg7, Lys8, Arg17, Arg27, and Arg28), which are likely to be in direct contact with the lipid bilayer. This is supported by the comparison of the amino acid sequences in Figure 10. The truncated human enzyme also possesses cationic residues in the corresponding region, but they are fewer in number (Arg35, Arg56, and Arg60). Thus, the human enzyme may rely to a greater extent on the presence of the transmembrane domain in order to achieve a stable interaction with the lipid bilayer, as opposed to the bacterial enzyme, which lacks such a structure and is also in need of using other electron acceptors besides membrane-embedded ubiquinones.

It is, however, interesting to note that the distribution of the cationic residues in the α1-α2 microdomain does not seem highly conserved in the Class II DHODHs in Figure 10, spanning a wide evolutionary distance. This conclusion is in line with a recent comprehensive bioinformatics study by Sousa et al. [76]. None of the positively charged residues on the outer surface of the α1-α2 microdomain were pointed out as highly conserved in an alignment of 1062 Class II DHODH sequences, or the whole α1-α2 microdomain, was one of the least conserved regions of the enzymes. It might be that the N-terminal part of DHODH has evolved to match the lipid composition of its respective cellular environment. In eukaryotes, together with the mitochondrial location, Class II DHODH have acquired a transmembrane domain, that anchors their position to the outer IMM. What other functions and benefits this transmembrane domain might have, is still open for discovery.

## 4. Materials and Methods

### 4.1. Chemicals

1-palmitoyl-2-oleoyl-glycero-3-phosphocholine (POPC), 1′,3′-bis[1,2-dioleoyl-sn-glycero-3-phospho]-glycerol sodium salt (TOCL), 1-palmitoyl-2-oleoyl-sn-glycero-3-phosphoethanolamine (POPE), and 1-palmitoyl-2-oleoyl-sn-glycero-3-phospho-(1′-rac-glycerol) sodium salt (POPG) were purchased from Avanti Polar Lipids (Alabaster, AL, USA). Coenzyme Q_10_, Tris-HCl, trisodium citrate, NaCl, CaCl_2_, and D_2_O (>99%) were purchased from Sigma-Aldrich (Stockholm, Sweden) and used without further purification. Chain-deuterated 1-palmitoyl-2-oleoyl-d_63_-glycero-3-phosphocholine (d_63_-POPC) was synthesized by the Deuteration and Macromolecular Crystallography (DEMAX) [78] platform at the European Spallation Source (ESS), Lund, Sweden, as previously published [67]. Complex polar phospholipid mixtures (h_3_pol) were extracted and purified from cultures of *Candida glabrata* displaying increased sensitivity to Amphotericin B, using procedures described elsewhere [54,69]. Undoped 80 × 50 × 15 mm^3^ silicon single crystals, polished on the (111) face to a typical roughness of <3 Å, were purchased from Siltronix (Archamps, France). The silicon substrates were cleaned in an aqueous piranha solution (5:4:1 H_2_O/H_2_SO_4_/H_2_O_2_) for 15 min at 80 °C, followed by extensive rinsing with ultrapure water and UV/ozone cleaning with a Jelight 144 AX cleaner from Bridge Tronic (Costa Mesa, CA, USA) for 10 min.

### 4.2. Expression and Purification of Proteins

The proteins used in this study were produced as previously described [43]. Briefly, pET-26b plasmids bearing the cDNA for either *Hs*Δ29DHODH or *Ec*DHODH were transformed into *E. coli* TUNER(DE3) cells (Merck KGaA, Darmstadt, Germany). Bacteria were cultured in Terrific Broth (TB) to an OD_600_ of 0.6–0.8 units. At this point, the cultures were supplemented with 100 µM FMN (Sigma-Aldrich, Stockholm, Sweden) and protein expression was induced with 100 µM IPTG. Protein expression took place for 20 h at 18 °C. The cells were harvested by centrifugation and disrupted using a French pressure cell at 18,000 psig (Glen Mills Inc., Clifton, NJ, USA). The proteins were purified by immobilized metal ion affinity chromatography on nickel sepharose (HisTrap HP, Cytiva Life Sciences, Uppsala, Sweden) columns, removal of the His-tag and by size-exclusion chromatography using Superdex 200 pg (Cytiva Life Sciences, Uppsala, Sweden). *Hs*Δ29DHODH and *Ec*DHODH preparations used had specific activities of 97 U/mg and 100 U/mg respectively, as determined by previously described methods [43].

### 4.3. Thermal Shift Assays (TSA)

*Hs*Δ29DHODH and *Ec*DHODH preparations used in NR experiments were used for TSA by nano differential scanning fluorimetry (nanoDSF) experiments. Here, we tested protein stability and aggregation in the four contrasts used in the NR experiments as follows: D_2_O, H_2_O, silicon-matched water (CMSi), and water matched to an SLD of 4 (CM4). The final protein concentration was 0.2 mg/mL and we used the same buffer composition as in the NR experiments, as described below.

The samples were loaded into a Prometheus NT.48 (NanoTemper Technologies GmbH, Munich, Germany) using standard grade capillaries. The samples were heated 1 °C/min, from 20 °C to 95 °C, and unfolding of the protein was analyzed with the ratio of the wavelengths measured at 350 nm and 330 nm (Tryptophan/Tyrosine shifts) and a laser power of 20%. From the resulting curves, the thermal unfolding transition midpoint T_m_ (°C), at which half of the protein population is unfolded, could be extracted.

### 4.4. Preparation of Small Unilamellar Vesicles

Lipid stocks, dissolved in a mixture of chloroform/methanol (9:1 *v*/*v*), were mixed in glass vials and dried under a stream of nitrogen. The resulting dry lipid film was resuspended, in either ultrapure water (R = 18.2 MΩ) or buffer (10 mM Tris-HCl, 2 mM CaCl_2,_ 100 mM NaCl, pH 7.4), to a concentration of 0.5 mg/mL The lipid films were allowed to hydrate for at least 30 min at room temperature. Small unilamellar vesicles (SUVs) were prepared by sonicating the resuspended lipids with a Vibra-Cell VCX 130 tip sonicator from Sonics & Materials Inc. (Newtown, CT, USA) for 5 min at 35% amplitude with a 10 s on/off cycle until the solutions became visibly clear.

### 4.5. Neutron Reflectivity Measurements

Experiments were conducted on the D17 reflectometer at the Institut Laue-Langevin, Grenoble, France [79,80] and on the INTER reflectometer at the ISIS Neutron and Muon Source of the STFC Rutherford Appleton Laboratory, Didcot, UK [81,82]. Specular neutron reflection was measured on the D17 reflectometer using neutron wavelengths (λ) of 2–20 Å to record reflectivity profiles between 0.01 < Q < 0.25 Å−1, and a 1–20% variable wavelength resolution (dλ/λ), where Q = 4π sin θ/λ is the momentum transfer vector of the neutrons in the direction (z) perpendicular to the membrane–water interface, and the reflectivity R is the ratio of the reflected intensity I_R_ to the incident intensity I_0_. Two incident angles θ (0.7° and 3.0°) were used to obtain the full reflectivity profiles, with the background scattering subtracted from the 2D detector images. On the INTER reflectometer, the incidence angles used were 0.7° and 2.3° and reflectivity profiles were recorded using neutron wavelengths in the range from 1 to 16 Å, covering a Q range from 0.009 to 0.3 Å^−1^ and an angular resolution dθ/θ of 4%. The background was not subtracted from INTER data and was fitted as part of the data analysis.

The structure of the Si-SiO_2_ surface was characterized in D_2_O and H_2_O prior to the addition of lipids. The lipid bilayers were formed in situ by means of the vesicle fusion method, as described previously [45,48]. Briefly, 3 mL of the vesicle solutions were injected immediately after sonication into the neutron reflectivity cells previously equilibrated at 30 °C. The vesicles were incubated for 30 min to allow then to fuse and spread over the crystal surface. After rinsing off the excess vesicles with buffer (10 mM Tris, 100 mM NaCl, pH or pD = 7.4) containing no CaCl_2_, each lipid membrane was characterized in the following four contrasts: D_2_O, H_2_O, silicon-matched water (CMSi), and water matched to a value of 4 × 10^−6^ Å^−2^ (CM4). The CMSi and CM4 contrasts were produced by mixing 38 vol% D_2_O and 62 vol% H_2_O, and 66 vol% D_2_O and 34 vol% H_2_O, respectively. Contrast changes were achieved by rinsing with 20 mL of the solvent using a Knauer 4P HPLC pump (KNAUER Wissenschaftliche Geräte GmbH, Berlin, Germany). Protein solutions (3 mL) in 10 mM Tris, 100 mM NaCl, and either pD or pH = 7.4, were injected over the lipid membranes at either 0.4 mg/mL, or stepwise at a concentration of 0.4 mg/mL and changes in reflectivity were recorded after a 30 min incubation period. The protein–lipid bilayers were characterized in each of the four contrasts (D_2_O, H_2_O, CM4, CMSi) after protein addition and after rinsing with buffer.

### 4.6. Data Evaluation

The neutron scattering length density profile [ρ(z)] of a lipid bilayer can be described by distinct regions corresponding to the polar head groups and hydrophobic acyl chains due to their chemical differences. Reflectivity analysis is based on modelling the thickness, scattering length density, solvent volume fraction, and interfacial roughness of typically three layers corresponding to the two lipid head groups and the central acyl chain region. In membranes containing lipids, water, and a protein, the scattering length density (ρ_layer_) is the sum of the molecular scattering length densities of the components ρ_i_ weighted by their volume fractions ϕ_i_ as follows:

ρ_layer_ = ϕ_lipid_ρ_lipid_ + ϕ_water_ρ_water_ + ϕ_protein_ρ_protein_

Thus, when there are significant differences in the molecular scattering length densities of lipid, protein, and water, their volume fractions can be computed from the fitted scattering length density profile of the membrane by using contrast variation. Similarly, in the selective deuteration of one lipid in a binary mixture can be used to determine the lipid composition in the bilayer, and the distribution (symmetric/asymmetric) of the two lipids. We used multidimensional contrast variation of both lipids (deuterated and non-deuterated chains) and the buffer solutions to obtain several different neutron data sets of each membrane, which were analyzed simultaneously, maintaining constant structural parameters (thickness, solvent fraction, roughness) that are unaffected by the degree of deuteration. Varying the solvent D_2_O content allows the solvent volume fraction in the membrane to be determined, in addition to the lipid composition and ubiquinone location to be revealed by the lipid contrast variation. The Motofit program [83], an extension of the IGOR Pro analysis package (Wavemetrics, Lake Oswego, OR, USA), was used for optical matrix modelling using the Abeles method [84] in order to calculate neutron reflectivity from the lipid/protein membranes and to refine the model until the best fit to the experimental data was achieved. The quality of the fits was judged by the agreement between the curves derived from the model and the measured data points, whilst attempting to minimize the global chi-squared value and using the constraints that, whenever possible, the solvent content in the lipid chain region was kept constant across different contrasts. The area per lipid molecule of the bilayer was constrained to be equal in the lipid headgroups and chains in each monolayer containing only lipids.

The sensitivity of each solvent contrast to each lipid contrast depends on the relative SLD differences, which gives a different fitting uncertainty for each parameter in different contrasts. The global fit and uncertainty are determined by the most sensitive contrast for each parameter, and these values are given in the results tables. Uncertainties in the fitted parameters (thickness, solvent fraction, SLD) were propagated to the calculated parameters (A_wet_, mol% of lipids/Q_10_/protein).

## 5. Conclusions

We have investigated two Class II DHODH, *Hs*Δ29DHODH, and *Ec*DHODH, in situ by neutron reflectometry and show, for the first time, the structural basis of their interaction with lipid membranes. Both of the enzymes interact with ubiquinone, that is exclusively located at the center of the lipid bilayers, by penetration into the outer membrane leaflet, without any detectable ubiquinone migration. The enzyme relative binding strength to the lipid bilayer and the degree of penetration depend on both the enzyme and the lipid composition of the bilayer, with cardiolipin, phosphatidylethanolamine, and phosphatidylglycerol, as well as other lipids present in mitochondria, promoting the interaction. This is, to our knowledge, the first time the membrane penetration of the enzymes lacking a transmembrane domain has been observed and points to a larger role of the alpha-helical domain in the membrane interaction than was previously thought. This initial study lays the foundation for further investigations probing the role of lipid headgroup and acyl chain composition, as well as mutations in the enzymes on the membrane interaction and enzyme activity.

## Figures and Tables

**Table 1:** Parameters corresponding to the best fits to the data from d_63_-POPC/TOCL membranes before and after addition of *Hs*Δ29DHODH, as displayed in Figure 2. τ = layer thickness, ρ = coherent neutron scattering length density (SLD) of the layers without the solvent contribution, φ = solvent volume fraction, σ = σ-value of a gaussian interfacial roughness between each layer and the previous layer. For simplicity, only the parameters corresponding to the inner protein layer are displayed. Fitting uncertainties are given for the most sensitive contrast. Detailed information is provided in Table S4.

**Lipid Bilayer**					
Layer	τ (Å)	ρ (10^−6^ Å^−2^) in D_2_O/CM4/CMSi/H_2_O	φ (vol%)	σ (Å)	vol% TOCL ^a^
Inner lipid heads	11 ± 1	2.0 ± 0.2	54 ± 5	3 ± 1	11 ± 2
Inner lipid chains	16 ± 1	5.4 ± 0.1	19 ± 3	4 ± 1	14 ± 2
Outer lipid chains	16 ± 1	4.6 ± 0.1	19 ± 3	2 ± 1	27 ± 2
Outer lipid heads	9 ± 1	2.1 ± 0.2	51 ± 5	7 ± 1	23 ± 2
**Lipid Bilayer + Protein**					
Layer	τ (Å)	ρ (10^−6^ Å^−2^) in D_2_O/CM4/CMSi/H_2_O	φ (vol%)	σ (Å)	vol% DHODH
Inner lipid heads	10 ± 1	2.0 ± 0.2	54 ± 5	3 ± 1	
Inner lipid chains	15 ± 1	5.4 ± 0.1	21 ± 3 ^b^	4 ± 1	
Outer chains + protein	15 ± 1	4.0/3.8/3.7/3.5 ± 0.1	21 ± 3 ^b^	4 ± 1	37 ± 8 ^c^
Outer heads + protein	8 ± 1	2.5/2.3/2.1/1.9 ± 0.5	44 ± 5	4 ± 1	51 ± 22 ^c^
Inner protein layer	43 ± 5	3.0/2.6/2.2/1.8 ± 0.2	84 ± 3 ^d^	5 ± 1	16 ± 2 ^e^

^a^ Relative to d_63_-POPC. ^b^ 21 ± 3% in D_2_O, CM4, and CMSi, 13 ± 3% in H_2_O. ^c^ Relative to the lipids. ^d^ 84 ± 3% in D_2_O, 90 ± 3% in CM4, 84 ± 50% in CMSi and 93 ± 3% in H_2_O. ^e^ Relative to water.

**Table 2:** Parameters corresponding to the best fits to the data from POPC/TOCL membranes before and after addition of *Ec*DHODH, as displayed in Figure 3. For simplicity, only the parameters corresponding to the inner protein layer are displayed. Fitting uncertainties are given for the most sensitive contrast. Detailed information is provided in Table S6.

**Lipid Bilayer**					
Layer	τ (Å)	ρ (10^−6^ Å^−2^) in D_2_O/CM4/CMSi/H_2_O	φ (vol%)	σ (Å)	vol% TOCL ^a^
Inner lipid heads	8 ± 1	2.0 ± 0.2	43 ± 8	3 ± 1	11 ± 2
Inner lipid chains	15 ± 1	−0.27 ± 0.1	10 ± 2	3 ± 1	14 ± 2
Outer lipid chains	15 ± 1	−0.27 ± 0.1	10 ± 2	3 ± 1	27 ± 2
Outer lipid heads	9 ± 1	2.1 ± 0.2	59 ± 8	6 ± 1	23 ± 2
**Lipid Bilayer + Protein**					
Layer	τ (Å)	ρ (10^−6^ Å^−2^) in D_2_O/CM4/CMSi/H_2_O	φ (vol%)	σ (Å)	vol% DHODH
Inner lipid heads	9 ± 1	2.0 ± 0.2	50 ± 8	4 ± 1	
Inner lipid chains	15 ± 1	−0.27 ± 0.1	19 ± 2	4 ± 1	
Outer chains + protein	15 ± 1	0.0025/−0.035/−0.063/0.11 ± 0.1	19 ± 2	5 ± 1	8 ± 3 ^b^
Outer heads + protein	9 ± 1	2.1 ± 0.5	50 ± 5	3 ± 1	7 ± 18 ^b^
Inner protein layer	46 ± 5	3.0/2.6/2.2/1.8 ± 0.2	72 ± 2 ^c^	3 ± 1	28 ± 3 ^d^

^a^ Relative to POPC. ^b^ Relative to the lipids. ^c^ 72 ± 2% in D_2_O, 77 ± 2% in CM4, 77 ± 50% in CMSi, and 81 ± 2% in H_2_O. ^d^ Relative to water.

**Table 3:** Parameters corresponding to the best fits to the data from d_63_-POPC/TOCL/Q_10_ membranes before and after addition of *Hs*Δ29DHODH, as displayed in Figure 4. For simplicity, only the parameters corresponding to the inner protein layer are displayed. Fitting uncertainties are given for the most sensitive contrast. Detailed information is provided in Table S7.

**Lipid Bilayer**					
Layer	τ (Å)	ρ (10^−6^ Å^−2^)	φ (vol%)	σ (Å)	vol%
Inner lipid heads	10 ± 1	2.0 ± 0.2	58 ± 5	3 ± 1	11 ± 3% TOCL ^a^
Inner lipid chains	13 ± 1	5.4 ± 0.2	9 ± 2	2 ± 1	14 ± 3% TOCL ^a^
Ubiquinone + chains	4 ± 1	2.7 ± 0.2	9 ± 2	1 ± 1	51 ± 5% Q10 ^b^
Outer lipid chains	13 ± 1	4.4 ± 0.2	9 ± 2	4 ± 1	29 ± 3% TOCL ^a^
Outer lipid heads	9 ± 1	2.1 ± 0.2	47 ± 5	5 ± 1	23 ± 3% TOCL ^a^
**Bilayer + Protein**					
Layer	τ (Å)	ρ (10^−6^ Å^−2^) in D_2_O/CM4/CMSi/H_2_O	φ (vol%)	σ (Å)	vol%
Inner lipid heads	11 ± 1	2.0 ± 0.2	48 ± 5	3 ± 1	
Inner lipid chains	13 ± 1	5.4 ± 0.2 ^c^	11 ± 2 ^d^	3 ± 1	
Ubiquinone + chains	4 ± 1	2.7 ± 0.2	11 ± 2 ^d^	2 ± 1	51 ± 5% Q10 ^b^
Outer chains + protein	13 ± 1	4.0/3.9/3.8/4.0 ± 0.2	11 ± 2 ^d^	2 ± 1	29 ± 14% DHODH ^b^
Outer heads + protein	8 ± 1	2.5/2.3/2.1/2.0 ± 0.5	40 ± 5	3 ± 1	42 ± 14% DHODH ^b^
Inner protein layer	36 ± 5	3.0/2.6/2.2/1.8 ± 0.2	70 ± 2 ^e^	4 ± 1	30 ± 2% DHODH ^f^

^a^ Relative to POPC. ^b^ Relative to the lipids. ^c^ 5.0 ± 0.2 × 10^−6^ Å^−2^ in H_2_O. ^d^ 0 ± 2 vol% in H_2_O. ^e^ 70 ± 2% in D_2_O, 76 ± 2% in CM4, 78 ± 20% in CMSi and 78 ± 2% in H_2_O. ^f^ Relative to water.

**Table 4:** Parameters corresponding to the best fits to the data from POPC/TOCL/Q_10_ before and after addition of *Ec*DHODH, as displayed in Figure 5. For simplicity, only the parameters corresponding to the inner protein layer are displayed. Fitting uncertainties are given for the most sensitive contrast. Detailed information is provided in Table S9.

Lipid Bilayer *					
Layer	τ (Å)	ρ (10^−6^ Å^−2^)	φ (vol%)	σ (Å)	vol%
Inner lipid heads	10 ± 1	2.0 ± 0.2	51 ± 8	3 ± 1	11 ± 3% TOCL ^a^
Inner lipid chains	14 ± 1	−0.27 ± 0.1	2 ± 2	3 ± 1	14 ± 3% TOCL ^a^
Ubiquinone + chains	4 ± 1	0.12 ± 0.12	2 ± 2	1 ± 1	51 ± 16% Q_10_ ^b^
Outer lipid chains	14 ± 1	−0.27 ± 0.1	2 ± 2	3 ± 1	29 ± 3% TOCL ^a^
Outer lipid heads	8 ± 1	2.1 ± 0.2	45 ± 10	4 ± 1	23 ± 3% TOCL ^a^
**Bilayer + Protein**					
Layer	τ (Å)	ρ (10^−6^ Å^−2^) in D_2_O/CM4/CMSi/H_2_O	φ (vol%)	σ (Å)	vol%
Inner lipid heads	10 ± 1	2.0 ± 0.2	56 ± 8	3 ± 1	11 ± 3% TOCL ^a^
Inner lipid chains	13 ± 1	−0.27 ± 0.1	10 ± 2	2 ± 1	14 ± 3% TOCL ^a^
Ubiquinone + chains	4 ± 1	0.12 ± 0.12	10 ± 2	1 ± 1	51 ± 16% Q_10_ ^b^
Outer chains + protein	13 ± 1	0.057/0.017/−0.023/−0.063 ± 0.2	10 ± 2	2 ± 1	10 ± 8% DHODH ^b^
Outer heads + protein	7 ± 1	2.3/2.2/2.1/2.1 ± 0.2	50 ± 5	4 ± 1	21 ± 12% DHODH ^b^
Inner protein layer	39 ± 5	3.0/2.6/2.2/1.8 ± 0.2	81 ± 3 ^c^	5 ± 1	19 ± 3% DHODH ^d^

* An additional layer 46 Å thick and separated by a 40 Å thick water layer was found floating on top of the lipid bilayer in the first contrast measured (D2O). This is likely to be a floating lipid bilayer on top of the supported lipid bilayer. ^a^ Relative to POPC. ^b^ Relative to the lipids. ^c^ 81 ± 3% in D_2_O, 81 ± 12% in CM4, 81 ± 50% in CMSi, and 81 ± 2% in H_2_O. ^d^ Relative to water.

**Table 5:** Parameters corresponding to the best fits to the data from *Candida glabrata* membranes before and after addition of *Hs*Δ29DHODH, as displayed in Figure 6. For simplicity, only the parameters corresponding to the inner protein layer are displayed. Fitting uncertainties are given for the most sensitive contrast. Detailed information is provided in Table S10.

**Lipid Bilayer**					
Layer	τ (Å)	ρ (10^−6^ Å^−2^)	φ (vol%)	σ (Å)	
Inner lipid heads	9 ± 1	3.0/2.8/2.6/2.4 ± 0.2	45 ± 5	4 ± 1	
Inner lipid chains	14 ± 1	−0.22 ± 0.1	2 ± 2	4 ± 1	
Outer lipid chains	14 ± 1	−0.22 ± 0.1	2 ± 2	3 ± 1	
Outer lipid heads	8 ± 1	3.0/2.8/2.6/2.4 ± 0.2	42 ± 5	3 ± 1	
**Bilayer + Protein**					
Layer	τ (Å)	ρ (10^−6^ Å^−2^) in D_2_O/CM4/CMSi/H_2_O	φ (vol%)	σ (Å)	vol% DHODH
Inner lipid heads	9 ± 1	3.0/2.8/2.6/2.4 ± 0.2	54 ± 5	4 ± 1	
Inner lipid chains	14 ± 1	−0.22 ± 0.1	15 ± 2	4 ± 1	
Outer chains + protein	14 ± 1	0.10/0.06/0.02/−0.02 ± 0.1	15 ± 2	4 ± 1	10 ± 5 ^a^
Outer heads + protein	8 ± 1	3.0/2.8/2.5/2.3 ± 0.2	50 ± 5	3 ± 1	18 ± 8 ^a^
Inner protein layer	35 ± 5	3.0/2.6/2.2/1.8 ± 0.2	70 ± 2 ^b^	3 ± 1	30 ± 2 ^c^

^a^ Relative to the lipids. ^b^ 91 ± 3% in H_2_O, 97 ± 3% in D_2_O, 70 ± 50% in CMSi, and 70 ± 2% in CM4. ^c^ Relative to water.

**Table 6:** Parameters corresponding to the best fits to the data from *Candida glabrata* bilayers supplemented with Q_10_ before and after addition of *Hs*Δ29DHODH, as displayed in Figure 7. For simplicity, only the parameters corresponding to the inner protein layer are displayed. Fitting uncertainties are given for the most sensitive contrast. Detailed information is provided in Table S11.

**Lipid Bilayer**					
Layer	τ (Å)	ρ (10^−6^ Å^−2^)	φ (vol%)	σ (Å)	vol% Q_10_
Inner lipid heads	9 ± 1	3.0/2.8/2.6/2.4 ± 0.2	46 ± 5	3 ± 1	
Inner lipid chains	13 ± 1	−0.22 ± 0.1	2 ± 2	4 ± 1	
Ubiquinone layer	4 ± 1	0.19 ± 0.1	2 ± 2	1 ± 1	57 ± 14 ^a^
Outer lipid chains	13 ± 1	−0.22 ± 0.1	2 ± 2	1 ± 1	
Outer lipid heads	9 ± 1	3.0/2.8/2.6/2.4 ± 0.2	49 ± 5	3 ± 1	
**Bilayer + Protein**					
Layer	τ (Å)	ρ (10^−6^ Å^−2^) in D_2_O/CM4/CMSi/H_2_O	φ (vol%)	σ (Å)	vol% DHODH
Inner lipid heads	9 ± 1	3.0/2.8/2.6/2.4 ± 0.2	53 ± 5	5 ± 1	
Inner lipid chains	13 ± 1	−0.22 ± 0.1	10 ± 2	4 ± 1	
Ubiquinone layer	4 ± 1	0.19 ± 0.1	10 ± 2	1 ± 1	
Outer chains + protein	13 ± 1	0.10/0.06/0.02/−0.02 ± 0.1	10 ± 2	1 ± 1	10 ± 5 ^a^
Outer heads + protein	8 ± 1	3.0/2.8/2.5/2.3 ± 0.2	50 ± 5	4 ± 1	18 ± 8 ^a^
Inner protein layer	40 ± 5	3.0/2.6/2.2/1.8 ± 0.2	70 ± 3 ^b^	6 ± 1	30 ± 3 ^c^

^a^ Relative to the lipids. ^b^ 82 ± 3% in H_2_O, 88 ± 3% in D_2_O, 82 ± 50% in CMSi, and 70 ± 3% in CM4. ^c^ Relative to water.

**Table 7:** Parameters corresponding to the best fits to the data from bacterial mimic membranes before and after addition of *Ec*DHODH, as displayed in Figure 8. For simplicity, only the parameters corresponding to the inner protein layer are displayed. Fitting uncertainties are given for the most sensitive contrast. Detailed information is provided in Table S12.

**Lipid Bilayer**					
Layer	τ (Å)	ρ (10^−6^ Å^−2^)	φ (vol%)	σ (Å)	
Inner lipid heads	9 ± 1	2.9/2.7/2.6/2.4 ± 0.2	56 ± 8	4 ± 1	
Inner lipid chains	16 ± 1	−0.27 ± 0.1	9 ± 2	6 ± 1	
Outer lipid chains	16 ± 1	−0.27 ± 0.1	9 ± 2	3 ± 1	
Outer lipid heads	9 ± 1	2.9/2.7/2.6/2.4 ± 0.2	56 ± 8	6 ± 1	
**Bilayer + Protein**					
Layer	τ (Å)	ρ (10^−6^ Å^−2^) in D_2_O/CM4/CMSi/H_2_O	φ (vol%)	σ (Å)	vol% DHODH
Inner lipid heads	9 ± 1	2.9/2.7/2.6/2.4 ± 0.2	60 ± 8	5 ± 1	
Inner lipid chains	16 ± 1	−0.27 ± 0.1 ^a^	16 ± 2 ^b^	6 ± 1	
Outer chains + protein	15 ± 1	0.55/0.45/0.35/0.11 ± 0.1	16 ± 2 ^b^	4 ± 1	25 ± 6 ^c^
Outer heads + protein	8 ± 1	2.9/2.7/2.5/2.3 ± 0.2	62 ± 5	5 ± 1	56 ± 8 ^c^
Inner protein layer	40 ± 5	3.0/2.6/2.2/1.8 ± 0.2	63 ± 2 ^d^	5 ± 1	37 ± 2 ^e^

^a^ 0.11 ± 0.1 × 10^−6^ Å^−2^ in H_2_O. ^b^ 0 ± 2 vol% for H_2_O. ^c^ Relative to the lipids. ^d^ 66 ± 3% in H_2_O, 75 ± 50% in CMSi, 70 ± 3% in CM4, and 63 ± 3% in D_2_O. ^e^ Relative to water.

## Supplementary Materials

Supplementary materials are available online at the following address: https://www.mdpi.com/article/10.3390/ijms23052437/s1.

## Accession Numbers

Full-length HsDHODH sequence (Uniprot Q02127, PYRD_HUMAN) and *E. coli* DHODH (Uniprot P0A7E1, PYRD_ECOLI).

## Abbreviations

*Proteins*	
*Hs*DHODH	human dihydroorotate dehydrogenase
*Hs*Δ29DHODH	human N-terminal truncated dihydroorotate dehydrogenase
*Ec*DHODH	dihydroorotate dehydrogenase from *Escherichia coli*
*Phospholipid classes*	
PC	phosphatidylcholine
CL	cardiolipin
PE	phosphatidylethanolamine
PG	phosphatidylglycerol
PS	phosphatidylserine
PI	phosphatidylinositol
*Synthetic lipids*	
POPC	1-palmitoyl,2-oleoyl-sn-glycero-3-phosphatidyl choline
d_63_-POPC	chain-deuterated POPC, 1-palmitoyl-*d*_31_, 2-oleoyl-*d*_32_-sn-glycero-3-phosphatidylcholine
TOCL	tetraoleoyl cardiolipin
POPE	1-palmitoyl,2-oleoyl-sn-glycero-3-phosphatidylethanolamine
POPG	1-palmitoyl,2-oleoyl-sn-glycero-3-phosphatidylglycerol
POPS	1-palmitoyl,2-oleoyl-sn-glycero-3-phosphatidylserine
POPI	1-palmitoyl,2-oleoyl-sn-glycero-3-phosphatidylinositol
*Other*	
Q_10_	ubiquinone Q_10_
IMM	inner mitochondrial membrane
SLD	scattering length density
CM4	buffer contrast-matched to SLD = 4.0 (66 vol% D_2_O and 32 vol% H_2_O)
CMSi	buffer contrast-matched to the silicon substrate SLD = 2.07 (38 vol% D_2_O and 62 vol% H_2_O)
ISIS	ISIS Pulsed Neutron and Muon Source, Rutherford Appleton Laboratory, Didcot OX11 0QX, United Kingdom
ILL	Institut Laue-Langevin, 71 Avenue des Martyrs, BP 156, 38042 Grenoble, France
