# Supplementary material for: New Insights into the Interaction of Class II Dihydroorotate Dehydrogenases with Ubiquinone in Lipid Bilayers as a Function of Lipid Composition
Source: Int J Mol Sci. 2022 Feb 23;23(5):2437. doi: 10.3390/ijms23052437 (PMC8910288; doi:10.3390/ijms23052437)
Supplement: Supplementary file 1 [file ijms-23-02437-s001.zip › ijms-1542747-supplementary.pdf]

SUPPLEMENTARY MATERIAL

New insights into the interaction of Class II dihydroorotate dehydrogenases with ubiquinone in lipid bilayers as a function of lipid composition

Juan Manuel Orozco Rodriguez[1], Hanna P. Wacklin-Knecht[2,3*], Luke A. Clifton[4], Oliver Bogojevic[3#], Anna Leung[3], Giovanna Fragneto[5], Wolfgang Knecht[1*]

[1] Department of Biology & Lund Protein Production Platform, Lund University, Sölvegatan 35, 22362 Lund, Sweden; JM.OR.: manuel.orozco@biol.lu.se; W.K.: wolfgang.knecht@biol.lu.se

[2] Department of Chemistry, Division of Physical Chemistry, Lund University, Naturvetarvägen 26, 22241 Lund, Sweden; hanna.wacklin-knecht@ess.eu

[3] European Spallation Source ERIC, Box 176, 221 00 Lund, Sweden; H.WK.: hanna.wacklin-knecht@ess.eu; O.B.: olbo@bce.au.dk; A.L.: anna.leung@ess.eu

[4] ISIS Pulsed Neutron and Muon Source, Rutherford Appleton Laboratory, Didcot OX11 0QX, United Kingdom; luke.clifton@stfc.ac.uk

[5] Institut Laue-Langevin, 71 Avenue des Martyrs, BP 156, 38042 Grenoble, France; fragneto@ill.fr

* Correspondence: H.WK.: hanna.wacklin-knecht@ess.eu; W.K.: wolfgang.knecht@biol.lu.se

[#] Current address: Department of Biological and Chemical Engineering - Faculty of Technical Sciences, Aarhus University, Gustav Wieds Vej 10, 8000 Aarhus C, Denmark, email: olbo@bce.au.dk

Table S1. Overview about the NR measurements performed in this study.

Lipid bilayer (mol%)	DHODH		Instrument (Location)
POPC and 10% TOCL	*Hs*Δ29DHODH[a]	*Ec*DHODH[d]	INTER (ISIS)/D17 (ILL)
d_{63}-POPC and 10% TOCL	*Hs*Δ29DHODH[b]		INTER (ISIS)
POPC, 10% TOCL and 10% Q_{10}	*Hs*Δ29DHODH[a]	*Ec*DHODH[d]	INTER (ISIS) /D17 (ILL)
d_{63}-POPC, 10% TOCL and 10% Q_{10}	*Hs*Δ29DHODH[b]		INTER (ISIS)
hIMM mimic (52% PC, 27% PS, 14% PE, 4% PI and 3% CL)	*Hs*Δ29DHODH[c]		D17 (ILL)
hIMM mimic (52% PC, 27% PS, 14% PE, 4% PI and 3% CL) and 10% Q_{10}	*Hs*Δ29DHODH[c]		D17 (ILL)
Bacterial Mimic (40% POPC, 35% POPE, 13% POPG and 12% TOCL)		*Ec*DHODH[d]	D17 (ILL)

[a] Ref. [81]
[b] Ref. [82]
[c] Ref. [80]
[d] Ref. [79]

Table S2. Neutron scattering length densities and molecular volumes used in this study.

	**POPC**	**TOCL**	**POPE**	**POPG**	**POPS**	**Q_{10}**	***C. glabrata phospholipids***
V_{head} (Å^3) [a]	322 [61]	490 [62]	245 [63]	289 [64]	278 [65]	252[d]	305[h]
V_{chains} (Å^3) [b]	934 [61]	1890 [62]	934 [61]	934 [61]	934 [61]	1324[e]	942[h]
SLD_{head} (10^{-6} Å^{-2}) [c]	1.86	2.98 (D_2O)<br>2.91 (CM4)<br>2.85 (CMSi)<br>2.77 (H_2O)	4.03 (D_2O)<br>3.57 (CM4)<br>3.19 (CMSi)<br>2.68 (H_2O)	3.19 (D_2O)<br>2.95 (CM4)<br>2.75 (CMSi)<br>2.47 (H_2O)	4.39 (D_2O)<br>3.99 (CM4)<br>3.65 (CMSi)<br>3.20 (H_2O)	1.81	3.0[h] (D_2O)<br>2.8[h] (CM4)<br>2.6[h] (CMSi)<br>2.4[h] (H_2O)
SLD_{chains} (10^{-6} Å^{-2}) [c]	-0.28 (h)<br>6.35 (d)	-0.22	-0.28	-0.28	-0.28	0.250	-0.22[h]
SLD_{total} (10^{-6} Å^{-2})						0.5	
$SLD_{protein}$ (10^{-6} Å^{-2})	D_2O	CM4	CMSi	H_2O	M_w (g mol^{-1}) [g]	V_m (Å^3)	
***Hs*Δ29DHODH**	3.0	2.6	2.2	1.8	40 263	49 165	
***Ec*DHODH**	3.0	2.6	2.2	1.8	36 775	45 576	

[a] Volume of the lipid headgroups, including the carbonyl groups and first carbon.
[b] Volume of the lipid chains.
[c] Neutron scattering length density of the lipid headgroups and chains, calculated from the component volumes [61-65] and isotopic composition (d = d_{63}-POPC).
[d] Volume corresponding to Coenzyme Q_0 (2,3-dimethoxy-5-methyl-1,4-benzoquinone).
[e] Volume calculated by adding the volumes of 10 isoprene units.
[f] Protein scattering length density, calculated on the basis of amino acid sequence, amino acid volumes [66] and proton exchange with deuterated solvents.
[g] Protein molecular weight.
[h] Calculated from the molar composition of the complex lipid mixture.

Table S3. Thermal stability data determined by nanoDSF. The melting temperature (mean ± SD) calculated from 3 independent measurements is reported. The buffer in the different contrasts was 10 mM Tris-HCl pH (pD) 7.4, 100 mM NaCl.

**Contrast**	**T_m (°C)**	
	*Hs*Δ29DHODH	*Ec*DHODH
**H_2O**	51.5 ± 0.1	54.5 ± 2.1
**CMSi**	52.6 ± 0.2	55.5 ± 0.7
**CM4**	52.8 ± 0.1	54.7 ± 1.9
**D_2O**	52.7 ± 0.3	56.2 ± 1.0

Table S4. Parameters corresponding to the best fits to the data from d_{63}-POPC/TOCL membranes before and after addition of *Hs*Δ29DHODH, and after rinse, as displayed in Figure 2. τ = layer thickness, ϱ = coherent neutron scattering length density (SLD) of the layers without the solvent contribution, φ = solvent volume fraction, σ = σ-value of a gaussian interfacial roughness between each layer and the previous layer. Fitting uncertainties are given for the most sensitive contrast.

**Lipid Bilayer**					
Layer	τ (Å)	ρ (10^{-6} Å^{-2}) in D_2O/CM4/CMSi/H_2O	φ (vol%)	σ (Å)	vol% TOCL[a]
Inner lipid heads	11 ± 1	2.0 ± 0.2	54 ± 5	3 ± 1	11 ± 2
Inner lipid chains	16 ± 1	5.4 ± 0.1	19 ± 3	4 ± 1	14 ± 2
Outer lipid chains	16 ± 1	4.6 ± 0.1	19 ± 3	2 ± 1	27 ± 2
Outer lipid heads	9 ± 1	2.1 ± 0.2	51 ± 5	7 ± 1	23 ± 2
**Lipid Bilayer + Protein**					
Layer	τ (Å)	ρ (10^{-6} Å^{-2}) in D_2O/CM4/CMSi/H_2O	φ (vol%)	σ (Å)	vol% DHODH
Inner lipid heads	10 ± 1	2.0 ± 0.2	54 ± 5	3 ± 1	
Inner lipid chains	15 ± 1	5.4 ± 0.1	21 ± 3 [b]	4 ± 1	
Outer lipid chains + protein	15 ± 1	4.0/3.8/3.7/3.5 ± 0.1	21 ± 3 [b]	4 ± 1	37 ± 8 [c]
Outer lipid heads + protein	8 ± 1	2.5/2.3/2.1/1.9 ± 0.5	44 ± 5	4 ± 1	51 ± 22 [c]
Protein layer 1	43 ± 5	3.0/2.6/2.2/1.8 ± 0.2	84 ± 3 [d]	5 ± 1	16 ± 2 [e]
Protein layer 2	60 ± 15	3.0/2.6/2.2/1.8 ± 0.2	96 ± 3 [f]	8 ± 1	4 ± 2 [e]
**After Rinse**					
Layer	τ (Å)	ρ (10^{-6} Å^{-2}) in D_2O/CM4/CMSi/H_2O	φ (vol%)	σ (Å)	vol% DHODH
Inner lipid heads	10 ± 1	2.0 ± 0.2	54 ± 5	3 ± 1	
Inner lipid chains	15 ± 1	5.4 ± 0.1	21 ± 3 [g]	5 ± 1	
Outer lipid chains + protein	15 ± 1	4.0/3.8/3.7/3.5 ± 0.1	21 ± 3 [g]	3 ± 1	37 ± 8 [c]
Outer lipid heads + protein	8 ± 1	2.5/2.3/2.1/1.9 ± 0.5	46 ± 5	5 ± 1	51 ± 22 [c]
Protein layer 1	46 ± 5	3.0/2.6/2.2/1.8 ± 0.2	88 ± 3 [h]	6 ± 1	12 ± 2 [e]
Protein layer 2	85 ± 15	3.0/2.6/2.2/1.8 ± 0.2	98 ± 3 [i]	8 ± 1	2 ± 2 [e]

[a] Relative to d_{63}-POPC.
[b] 21 ± 3% in D_2O, CM4 and CMSi, 13 ± 3% in H_2O.
[c] Relative to the lipids.
[d] 84 ± 3% in D_2O, 90 ± 3% in CM4, 84 ± 50% in CMSi and 93 ± 3% in H_2O
[e] Relative to water.
[f] 96 ± 3% in D_2O and CM4, 96 ± 50% in CMSi, 100% ± 3% in H_2O.
[g] 21 ± 3% in D_2O, CM4 and CMSi, 16 ± 3% in H_2O.
[h] 88 ± 3% in D_2O, 94 ± 3% in CM4, 85 ± 50% in CMSi, 97 ± 3% in H_2O.
[i] 98 ± 3% in D_2O and CM4, 98 ± 50% in CMSi, 100 ± 3% in H_2O.

Table S5. Parameters corresponding to the best fits to the data from the POPC/TOCL bilayer before and after addition of *Hs*Δ29DHODH, and after rinse, as displayed in Fig. S1. Fitting uncertainties are given for the most sensitive contrast.

**Lipid Bilayer**					
Layer	τ (Å)	ρ (10^{-6} Å^{-2})	φ (vol%)	σ (Å)	vol% DHODH
Inner lipid heads	8 ± 1	2.0 ± 0.2	35 ± 5	3 ± 1	
Inner lipid chains	16 ± 1	-0.27 ± 0.1	9 ± 2	3 ± 1	
Outer lipid chains	16 ± 1	-0.27 ± 0.1	9 ± 2	3 ± 1	
Outer lipid heads	8 ± 1	2.1 ± 0.2	47 ± 5	6 ± 1	
**Bilayer + Protein**					
Layer	τ (Å)	ρ (10^{-6} Å^{-2}) in D_2O/H_2O	φ (vol%)	σ (Å)	vol% DHODH
Inner lipid heads	8 ± 1	2.0 ± 0.2	35 ± 7	4 ± 1	
Inner lipid chains	16 ± 1	-0.27 ± 0.1	9 ± 2	4 ± 1	
Outer lipid chains	16 ± 1	-0.27 ± 0.1	9 ± 2	3 ± 1	
Outer lipid heads	8 ± 1	2.1 ± 0.2	45 ± 5	5 ± 1	20 ± 22 [a]
Protein layer 1	46 ± 5	3.0/1.8 ± 0.2	93 ± 3 [b]	5 ± 1	7 ± 3 [c]
Protein layer 2	75 ± 15	3.0/1.8 ± 0.2	98 ± 3 [d]	10 ± 1	2 ± 3 [c]
**After Rinse**					
Layer	τ (Å)	ρ (10^{-6} Å^{-2}) in D_2O/H_2O	φ (vol%)	σ (Å)	vol% DHODH
Inner lipid heads	8 ± 1	2.0 ± 0.2	35 ± 5	4 ± 1	
Inner lipid chains	16 ± 1	-0.27 ± 0.1	9 ± 2	4 ± 1	
Outer lipid chains	16 ± 1	-0.27 ± 0.1	9 ± 2	3 ± 1	
Outer lipid heads	8 ± 1	2.1 ± 0.2	46 ±5	5 ± 1	20 ± 22 [a]
Protein layer 1	40 ± 5	3.0/1.8 ± 0.2	96 ± 3 [e]	5 ± 1	4 ± 3 [c]

[a] Relative to the lipids.
[b] 93 ± 4% in D_2O and 92 ± 3% in H_2O.
[c] Relative to water.
[d] 98 ± 4% in D_2O and 98 ± 3% in H_2O.
[e] 96 ± 4% in D_2O and 96 ± 3% in H_2O.

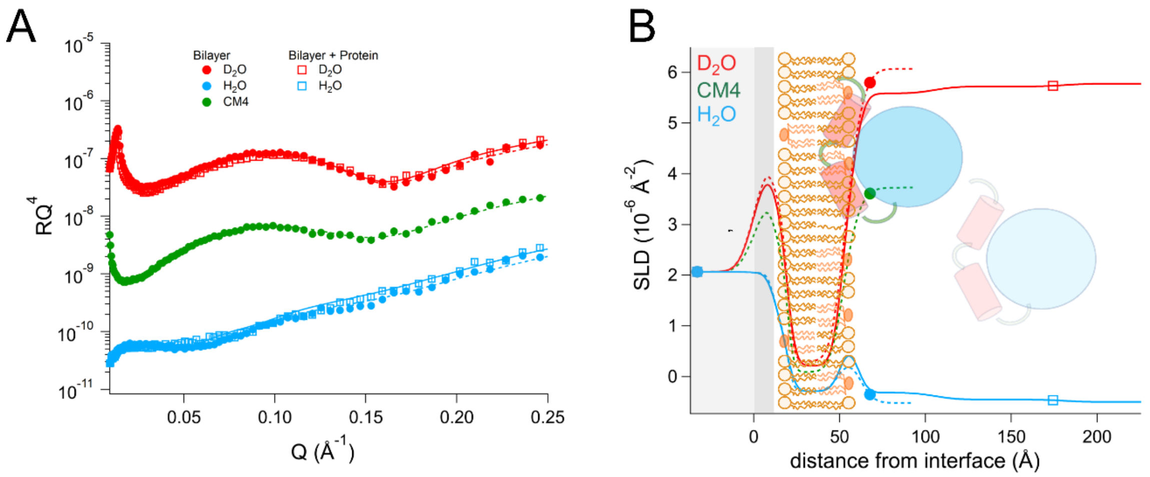

Figure S1. (A) Reflectivity curves (data from INTER, ISIS) and (B) SLD profile for POPC/TOCL bilayers before and after addition of *Hs*Δ29DHODH with a schematic representation of the model structure. POPC molecules are shown in brown (hollow heads, two tails). TOCL molecules are depicted in orange (filled heads, four tails). The α1-α2 microdomain of the protein is shown in red and the catalytic domain is depicted in blue.

Table S6. Parameters corresponding to the best fits to the data from POPC/TOCL membranes before and after addition of *Ec*DHODH, and after rinse, as displayed in Figure 3. Fitting uncertainties are given for the most sensitive contrast.

**Lipid Bilayer**					
Layer	τ (Å)	ρ (10^{-6} Å^{-2})	φ (vol%)	σ (Å)	vol% TOCL [a]
Inner lipid heads	8 ± 1	2.0 ± 0.2	43 ± 8	3 ± 1	11 ± 2
Inner lipid chains	15 ± 1	-0.27 ± 0.1	10 ± 2	3 ± 1	14 ± 2
Outer lipid chains	15 ± 1	-0.27 ± 0.1	10 ± 2	3 ± 1	27 ± 2
Outer lipid heads	9 ± 1	2.1 ± 0.2	59 ± 8	6 ± 1	23 ± 2
**Lipid Bilayer + Protein**					
Layer	τ (Å)	ρ (10^{-6} Å^{-2}) in D_2O/CM4/CMSi/H_2O	φ (vol%)	σ (Å)	vol% DHODH
Inner lipid heads	9 ± 1	2.0 ± 0.2	50 ± 8	4 ± 1	
Inner lipid chains	15 ± 1	-0.27 ± 0.1	19 ± 2	4 ± 1	
Outer chains + protein	15 ± 1	0.0025/-0.035/-0.063/0.11 ± 0.1	19 ± 2	5 ± 1	8 ± 3 [b]
Outer heads + protein	9 ± 1	2.1 ± 0.5	50 ± 5	3 ± 1	7 ± 18 [b]
Protein layer 1	46 ± 5	3.0/2.6/2.2/1.8 ± 0.2	72 ± 2 [c]	3 ± 1	28 ± 3 [d]
Protein layer 2	55 ± 15	3.0/2.6/2.2/1.8 ± 0.2	84 ± 3 [e]	10 ± 1	16 ± 3 [d]
Protein layer 3	55 ± 15	3.0/2.6/2.2/1.8 ± 0.2	92 ± 3 [f]	12 ± 1	8 ± 3 [d]
Protein layer 4	55 ± 15	3.0/2.6/2.2/1.8 ± 0.2	96 ± 3 [g]	12 ± 1	4 ± 3 [d]
**After rinse**					
Layer	τ (Å)	ρ (10^{-6} Å^{-2}) in D_2O/CM4/CMSi/H_2O	φ (vol%)	σ (Å)	vol% DHODH
Inner lipid heads	9 ± 1	2.0 ± 0.2	50 ± 8	4 ± 1	
Inner lipid chains	15 ± 1	-0.27 ± 0.1	21 ± 2	4 ± 1	
Outer chains + protein	15 ± 1	0.0025/-0.035/-0.063/0.11 ± 0.1	21 ± 2	4 ± 1	8 ± 3 [b]
Outer heads + protein	10 ± 1	2.1 ± 0.5	58 ± 5	4 ± 1	7 ± 18 [b]
Protein layer 1	46 ± 5	3.0/2.6/2.2/1.8 ± 0.2	75 ± 2 [h]	4 ± 1	25 ± 3 [d]
Protein layer 2	55 ± 15	3.0/2.6/2.2/1.8 ± 0.2	84 ± 3 [i]	8 ± 1	16 ± 3 [d]
Protein layer 3	55 ± 15	3.0/2.6/2.2/1.8 ± 0.2	91 ± 3 [j]	12 ± 1	9 ± 3 [d]
Protein layer 4	55 ± 15	3.0/2.6/2.2/1.8 ± 0.2	96 ± 3 [k]	12 ± 1	4 ± 3 [d]

[a] Relative to POPC.
[b] Relative to the lipids.
[c] 72 ± 2% in D_2O, 77 ± 2% in CM4, 77 ± 50% in CMSi and 81 ± 2% in H_2O.
[d] Relative to water.
[c] 84 ± 2% in D_2O, 87 ± 2% in CM4, 90 ± 50% in CMSi and 94 ± 2% in H_2O.
[f] 92 ± 2% in D_2O, 92 ± 2% in CM4, 92 ± 50% in CMSi and 97 ± 2% in H_2O.
[g] 96 ± 2% in D_2O, 97 ± 2% in CM4, 97 ± 50% in CMSi and 100 ± 2% in H_2O.
[h] 75 ± 2% in D_2O, 83 ± 2% in CM4, 85 ± 50% in CMSi and 89 ± 2% in H_2O.
[i] 84 ± 2% in D_2O, 87 ± 2% in CM4, 93 ± 50% in CMSi and 94 ± 2% in H_2O.
[j] 91 ± 2% in D_2O, 92 ± 2% in CM4, 92 ± 50% in CMSi and 97 ± 2% in H_2O.
[k] 96 ± 2% in D_2O, 96 ± 2% in CM4, 97 ± 50% in CMSi and 100 ± 2% in H_2O.

Table S7. Parameters corresponding to the best fits to the data from d_{63}-POPC/TOCL/Q_{10} membranes before and after addition of *Hs*Δ29DHODH, and after buffer rinse, as displayed in Figure 4. Fitting uncertainties are given for the most sensitive contrast.

Lipid Bilayer					
Layer	τ (Å)	ρ (10^{-6} Å^{-2})	φ (vol%)	σ (Å)	vol%
Inner lipid heads	10 ± 1	2.0 ± 0.2	58 ± 5	3 ± 1	11 ± 3% TOCL [a]
Inner lipid chains	13 ± 1	5.4 ± 0.2	9 ± 2	2 ± 1	14 ± 3% TOCL [a]
Ubiquinone + chains	4 ± 1	2.7 ± 0.2	9 ± 2	1 ± 1	51 ± 5% Q_{10} [b]
Outer lipid chains	13 ± 1	4.4 ± 0.2	9 ± 2	4 ± 1	29 ± 3% TOCL [a]
Outer lipid heads	9 ± 1	2.1 ± 0.2	47 ± 5	5 ± 1	23 ± 3% TOCL [a]
**Bilayer + Protein**					
Layer	τ (Å)	ρ (10^{-6} Å^{-2}) in D_2O/CM4/CMSi/H_2O	φ (vol%)	σ (Å)	vol%
Inner lipid heads	11 ± 1	2.0 ± 0.2	48 ± 5	3 ± 1	
Inner lipid chains	13 ± 1	5.4 ± 0.2 [c]	11 ± 2 [d]	3 ± 1	
Ubiquinone + chains	4 ± 1	2.7 ± 0.2	11 ± 2 [d]	2 ± 1	51 ± 5% Q_{10} [b]
Outer chains + protein	13 ± 1	4.0/3.9/3.8/4.0 ± 0.2	11 ± 2 [d]	2 ± 1	29 ± 14% DHODH [b]
Outer heads + protein	8 ± 1	2.5/2.3/2.1/2.0 ± 0.5	40 ± 5	3 ± 1	42 ± 14% DHODH [b]
Protein Layer 1	36 ± 5	3.0/2.6/2.2/1.8 ± 0.2	70 ± 2 [e]	4 ± 1	30 ± 2% DHODH [f]
Protein Layer 2	49 ± 15	3.0/2.6/2.2/1.8 ± 0.2	92 ± 2 [g]	5 ± 1	8 ± 2% DHODH [f]
Protein Layer 3	62 ± 15	3.0/2.6/2.2/1.8 ± 0.2	94 ± 2 [h]	10 ± 1	6 ± 2% DHODH [f]
**After Rinse**					
Layer	τ (Å)	ρ (10^{-6} Å^{-2}) in D_2O/CM4/CMSi/H_2O	φ (vol%)	σ (Å)	vol%
Inner lipid heads	10 ± 1	2.0 ± 0.2	55 ± 5	3 ± 1	
Inner lipid chains	12 ± 1	5.3 ± 0.2 [c]	13 ± 2 [d]	3 ± 1	
Ubiquinone + chains	4 ± 1	2.7 ± 0.2	13 ± 2 [d]	2 ± 1	51 ± 5% Q_{10} [b]
Outer chains + protein	12 ± 1	3.7/3.5/3.3/4.0 ± 0.2	13 ± 2 [d]	4 ± 1	50 ± 14% DHODH [b]
Outer heads + protein	8 ± 1	2.45/2.3/2.1/2.0 ± 0.5	51 ± 5	4 ± 1	38 ± 14% DHODH [b]
Protein Layer 1	38 ± 5	3.0/2.6/2.2/1.8 ± 0.2	80 ± 2 [i]	5 ± 1	20 ± 2% DHODH [f]
Protein Layer 2	55 ± 15	3.0/2.6/2.2/1.8 ± 0.2	96 ± 2 [j]	10 ± 1	4 ± 2% DHODH [f]
Protein Layer 3	55 ± 15	3.0/2.6/2.2/1.8 ± 0.2	95 ± 2 [k]	10 ± 1	5 ± 2% DHODH [f]

[a] Relative to POPC.
[b] Relative to the lipids.
[c] 5.0 ± 0.2 × 10^{-6} Å^{-2} in H_2O.
[d] 0 ± 2 vol% in H_2O.
[e] 70 ± 2% in D_2O, 76 ± 2% in CM4, 78 ± 20% in CMSi and 78 ± 2% in H_2O.
[f] Relative to water.
[g] 92 ± 2% in D_2O, 95 ± 2% in CM4, 95 ± 20% in CMSi and 96 ± 2% in H_2O.
[h] 94 ± 2% in D_2O, 93 ± 2% in CM4, 90 ± 20% in CMSi and 86 ± 2% in H_2O.
[i] 80 ± 2% in D_2O, 87 ± 2% in CM4, 88 ± 20% in CMSi and 87 ± 2% in H_2O.
[j] 96 ± 2% in D_2O, 96 ± 2% in CM4, 96 ± 20% in CMSi and 97 ± 2% in H_2O.
[k] 95 ± 2% in D_2O, 96 ± 2% in CM4, 93 ± 20% in CMSi and 92 ± 2% in H_2O.

Table S8. Parameters corresponding to the best fits to the data from POPC/TOCL/Q_{10} bilayers before and after addition of *Hs*Δ29DHODH, and after rinse, as displayed in Figure S2. Fitting uncertainties are given for the most sensitive contrast.

Layer	τ (Å)	ρ (10^{-6} Å^{-2})	φ (vol%)	σ (Å)	vol%
**Lipid Bilayer**					
Layer	τ (Å)	ρ (10^{-6} Å^{-2})	φ (vol%)	σ (Å)	vol%
Inner lipid heads	8 ± 1	2.0 ± 0.1	45 ± 8	4 ± 1	
Inner lipid chains	13.5 ± 1	-0.27 ± 0.05	5 ± 2	4 ± 1	
Ubiquinone + Chains	4 ± 1	0.12 ± 0.1	5 ± 2	2 ± 1	51 ± 13% Q_{10} [a]
Outer lipid chains	13.5 ± 1	-0.27 ± 0.05	5 ± 2	3 ± 1	
Outer lipid heads	9 ± 1	2.1 ± 0.1	52 ± 8	4 ± 1	
**Bilayer + Protein**					
Layer	τ (Å)	ρ (10^{-6} Å^{-2}) in D_2O/H_2O	φ (vol%)	σ (Å)	vol%
Inner lipid heads	8 ± 1	2.0 ± 0.1	45 ± 8	4 ± 1	
Inner lipid chains	13 ± 1	-0.27 ± 0.05	13 ± 2	4 ± 1	
Ubiquinone + chains	4 ± 1	0.12 ± 0.1	13 ± 2	1 ± 1	51 ± 13 vol% Q_{10} [a]
Outer chains + protein	13 ± 1	-0.17 ± 0.1	13 ± 2	3 ± 1	3 ± 3 vol% DHODH [a]
Outer heads + protein	8 ± 1	2.3/2.1 ± 0.2	45 ± 5	5 ± 1	20 ± 22% DHODH [a]
Protein layer 1	38 ± 5	3.0/1.8 ± 0.2	78 ± 2 [b]	5 ± 1	22 ± 2% DHODH [c]
Protein layer 2	50 ± 15	3.0/1.8 ± 0.2	89 ± 2 [d]	5 ± 1	11 ± 2% DHODH [c]
Protein layer 3	50 ± 15	3.0/1.8 ± 0.2	88 ± 2 [e]	10 ± 1	12 ± 2% DHODH [c]
**After Rinse**					
Layer	τ (Å)	ρ (10^{-6} Å^{-2}) in D_2O/H_2O	φ (vol%)	σ (Å)	vol%
Inner lipid heads	8 ± 1	2.0 ± 0.1	45 ± 8	4 ± 1	
Inner lipid chains	13 ± 1	-0.27 ± 0.05	14 ± 2	4 ± 1	
Ubiquinone + chains	4 ± 1	0.12 ± 0.1	14 ± 2	2 ± 1	51 ± 13 vol% Q_{10} [a]
Outer chains + protein	13 ± 1	-0.17 ± 0.1	14 ± 2	3 ± 1	3 ± 3 vol% DHODH [a]
Outer heads + protein	8 ± 1	2.3/2.1 ± 0.2	48 ± 5	5 ± 1	20 ± 22% DHODH [a]
Protein layer 1	38 ± 5	3.0/1.8 ± 0.2	88 ± 2 [f]	5 ± 1	12 ± 2% DHODH [c]
Protein layer 2	50 ± 15	3.0/1.8 ± 0.2	92 ± 2 [g]	5 ± 1	8 ± 2% DHODH [c]
Protein layer 3	50 ± 15	3.0/1.8 ± 0.2	89 ± 2 [h]	10 ± 1	11 ± 2% DHODH [c]

[a] Relative to the lipids.

[b] 78 ± 2% in D_2O, 88 ± 2% in H_2O.

[c] Relative to water.

[d] 89 ± 2% in D_2O, 94 ± 2% in H_2O.

[e] 88 ± 2% in D_2O, 97 ± 2% in H_2O.

[f] 88 ± 2% in D_2O, 90 ± 2% in H_2O.

[g] 92 ± 2% in D_2O, 96 ± 2% in H_2O.

[h] 89 ± 2% in D_2O, 98 ± 2% in H_2O.

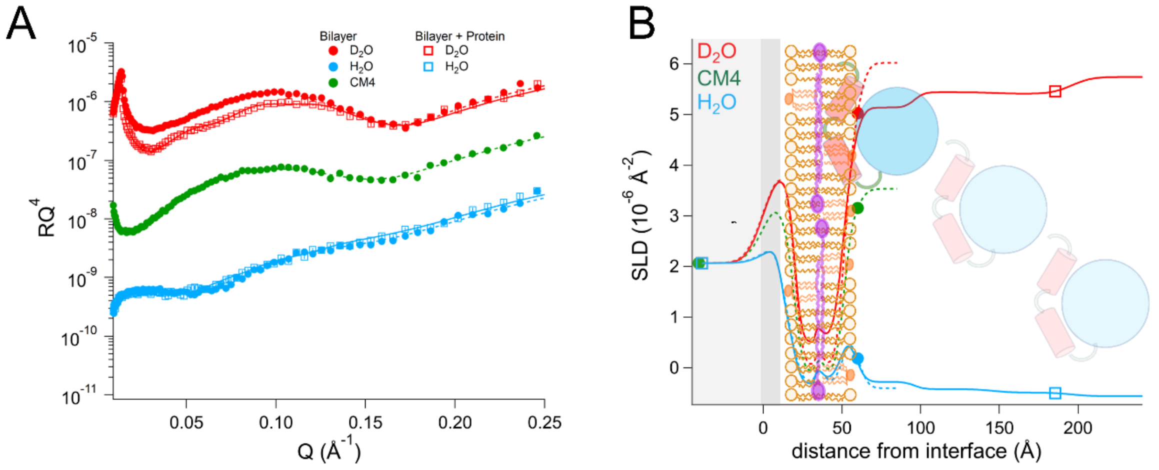

Figure S2. (A) Reflectivity curves (data from INTER, ISIS) and (B) SLD profile for POPC/TOCL/Q_{10} bilayers before and after addition of *Hs*Δ29DHODH with a schematic representation of the model structure. POPC molecules are shown in brown (hollow heads, two tails). TOCL molecules are depicted in orange (filled heads, four tails). The α1-α2 microdomain of the protein is shown in red and the catalytic domain is depicted in blue. Ubiquinone molecules are represented in purple (filled heads, long tails).

Table S9. Parameters corresponding to the best fits to the data from POPC/TOCL/Q_{10} before and after addition of *Ec*DHODH, and after rinse, as displayed in Fig. 5. Fitting uncertainties are given for the most sensitive contrast.

Lipid Bilayer					
Layer	τ (Å)	ρ (10^{-6} Å^{-2})	φ (vol%)	σ (Å)	vol%
Inner lipid heads	10 ± 1	2.0 ± 0.2	51 ± 8	3 ± 1	11 ± 3% TOCL [a]
Inner lipid chains	14 ± 1	-0.27 ± 0.1	2 ± 2	3 ± 1	14 ± 3% TOCL [a]
Ubiquinone + chains	4 ± 1	0.12 ± 0.12	2 ± 2	1 ± 1	51 ± 16% Q_{10} [b]
Outer lipid chains	14 ± 1	-0.27 ± 0.1	2 ± 2	3 ± 1	29 ± 3% TOCL [a]
Outer lipid heads	8 ± 1	2.1 ± 0.2	45 ± 10	4 ± 1	23 ± 3% TOCL [a]
**Bilayer + Protein**					
Layer	τ (Å)	ρ (10^{-6} Å^{-2}) in D_2O/CM4/CMSi/H_2O	φ (vol%)	σ (Å)	vol%
Inner lipid heads	10 ± 1	2.0 ± 0.2	56 ± 8	3 ± 1	11 ± 3% TOCL [a]
Inner lipid chains	13 ± 1	-0.27 ± 0.1	10 ± 2	2 ± 1	14 ± 3% TOCL [a]
Ubiquinone + chains	4 ± 1	0.12 ± 0.12	10 ± 2	1 ± 1	51 ± 16% Q_{10} [b]
Outer chains + protein	13 ± 1	0.057/0.017/-0.023/-0.063 ± 0.2	10 ± 2	2 ± 1	10 ± 8% DHODH [b]
Outer heads + protein	7 ± 1	2.3/2.2/2.1/2.1 ± 0.2	50 ± 5	4 ± 1	21 ± 12% DHODH [b]
Protein layer 1	39 ± 5	3.0/2.6/2.2/1.8 ± 0.2	81 ± 3 [c]	5 ± 1	19 ± 3% DHODH [d]
Protein layer 2	73 ± 15	3.0/2.6/2.2/1.8 ± 0.2	97 ± 3 [e]	10 ± 1	3 ±3% DHODH [d]
**After Rinse**					
Layer	τ (Å)	ρ (10^{-6} Å^{-2}) in D_2O/CM4/CMSi/H_2O	φ (vol%)	σ (Å)	vol%
Inner lipid heads	10 ± 1	2.0 ± 0.2	57 ± 8	3 ± 1	11 ± 3% TOCL [a]
Inner lipid chains	13 ± 1	-0.27 ± 0.1	10 ± 2	2 ± 1	14 ± 3% TOCL [a]
Ubiquinone + Chains	4 ± 1	0.12 ± 0.12	10 ± 2	1 ± 1	51 ± 16% Q_{10} [b]
Outer chains + protein	13 ± 1	0.057/0.017/-0.023/-0.063 ± 0.2	10 ± 2	2 ± 1	10 ± 8% DHODH [b]
Outer heads + protein	7 ± 1	2.3/2.2/2.1/2.1 ± 0.2	52 ± 5	3 ± 1	21 ± 12% DHODH [b]
Protein layer 1	39 ± 5	3.0/2.6/2.2/1.8 ± 0.2	81 ± 3 [f]	4 ± 1	19 ± 3% DHODH [d]
Protein layer 2	73 ± 15	3.0/2.6/2.2/1.8 ± 0.2	97 ± 3 [g]	8 ± 1	3 ±3% DHODH [d]

*An additional layer 46 Å thick and separated by a 40 Å thick water layer was found floating on top of the lipid bilayer in the first contrast measured (D_2O). This is likely to be a floating lipid bilayer on top of the supported lipid bilayer.

[a] Relative to POPC.

[b] Relative to the lipids.

[c] 81 ± 3% in D_2O, 81 ± 12% in CM4, 81 ± 50% in CMSi and 81 ± 2% in H_2O.

[d] Relative to water.

[e] 97 ± 3% in D_2O, 97 ± 12% in CM4, 97 ± 50% in CMSi and 97 ± 2% in H_2O.

[f] 81 ± 4% in D_2O, 81 ± 7% in CM4, 81 ± 50% in CMSi and 85 ± 3% in H_2O.

[g] 97 ± 4% in D_2O, 97 ± 7% in CM4, 97 ± 50% in CMSi and 97 ± 3% in H_2O.

Table S10. Parameters corresponding to the best fits to the data from *Candida glabrata* membranes before and after addition of *Hs*Δ29DHODH, and after rinse, as displayed in Figure 6. Fitting uncertainties are given for the most sensitive contrast.

**Lipid Bilayer**					
Layer	τ (Å)	ρ (10^{-6} Å^{-2})	φ (vol%)	σ (Å)	
Inner lipid heads	9 ± 1	3.0/2.8/2.6/2.4 ± 0.2	45 ± 5	4 ± 1	
Inner lipid chains	14 ± 1	-0.22 ± 0.1	2 ± 2	4 ± 1	
Outer lipid chains	14 ± 1	-0.22 ± 0.1	2 ± 2	3 ± 1	
Outer lipid heads	8 ± 1	3.0/2.8/2.6/2.4 ± 0.2	42 ± 5	3 ± 1	
**Bilayer + Protein**					
Layer	τ (Å)	ρ (10^{-6} Å^{-2}) in D_2O/CM4/CMSi/H_2O	φ (vol%)	σ (Å)	vol% DHODH
Inner lipid heads	9 ± 1	3.0/2.8/2.6/2.4 ± 0.2	54 ± 5	4 ± 1	
Inner lipid chains	14 ± 1	-0.22 ± 0.1	15 ± 2	4 ± 1	
Outer chains + protein	14 ± 1	0.10/0.06/0.02/-0.02 ± 0.1	15 ± 2	4 ± 1	10 ± 5 [a]
Outer heads + protein	8 ± 1	3.0/2.8/2.5/2.3 ± 0.2	50 ± 5	3 ± 1	18 ± 8 [a]
Protein layer 1	35 ± 5	3.0/2.6/2.2/1.8 ± 0.2	70 ± 2[b]	3 ± 1	30 ± 2 [c]
Protein layer 2	75 ± 15	3.0/2.6/2.2/1.8 ± 0.2	84 ± 2[d]	10 ± 1	16 ± 2 [c]
Protein layer 3	60 ± 15	3.0/2.6/2.2/1.8 ± 0.2	94 ± 2[e]	10 ± 1	6 ± 2 [c]
Protein layer 4	65 ± 15	3.0/2.6/2.2/1.8 ± 0.2	94 ± 2[f]	10 ± 1	6 ± 2 [c]
Protein layer 5	65 ± 15	3.0/2.6/2.2/1.8 ± 0.2	96 ± 2[g]	10 ± 1	4 ± 2 [c]
**After Rinse**					
Layer	τ (Å)	ρ (10^{-6} Å^{-2}) in D_2O/CM4/CMSi/H_2O	φ (vol%)	σ (Å)	vol% DHODH
Inner lipid heads	9 ± 1	3.0/2.8/2.6/2.4 ± 0.2	59 ± 3	4 ± 1	
Inner lipid chains	14 ± 1	-0.22 ± 0.1	18 ± 1	4 ± 1	
Outer chains + protein	14 ± 1	0.10/0.06/0.02/-0.02 ± 0.1	18 ± 1	3 ± 1	10 ± 5 [a]
Outer heads + protein	8 ± 1	3.0/2.8/2.5/2.3 ± 0.2	50 ± 3	3 ± 1	18 ± 8 [a]
Protein layer 1	40 ± 5	3.0/2.6/2.2/1.8 ± 0.2	80 ± 2[h]	3 ± 1	20 ± 2 [c]
Protein layer 2	60 ± 15	3.0/2.6/2.2/1.8 ± 0.2	87 ± 2[i]	4 ± 1	13 ± 2 [c]
Protein layer 3	60 ± 15	3.0/2.6/2.2/1.8 ± 0.2	91 ± 2[j]	10 ± 1	9 ± 2 [c]
Protein layer 4	60 ± 15	3.0/2.6/2.2/1.8 ± 0.2	92 ± 2[k]	10 ± 1	8 ± 2 [c]
Protein layer 5	70 ± 15	3.0/2.6/2.2/1.8 ± 0.2	95 ± 2[l]	10 ± 1	5 ± 2 [c]

[a] Relative to the lipids.
[b] 91 ± 3% in H_2O, 97 ± 3% in D_2O, 70 ± 50% in CMSi, 70 ± 3% in CM4.
[c] Relative to water.
[d] 95 ± 3% in H_2O, 93 ± 3% in D_2O, 96 ± 50% in CMSi, 84 ± 3% in CM4.
[e] 96 ± 3% in H_2O, 97 ± 3% in D_2O, 98 ± 50% in CMSi, 94 ± 3% in CM4.
[f] 98 ± 3% in H_2O, 98 ± 3% in D_2O, 98 ± 50% in CMSi, 94 ± 3% in CM4.
[g] 100 ± 3% in H_2O, 100 ± 3% in D_2O, 100 ± 50% in CMSi, 96 ± 3% in CM4.
[h] 94 ± 3% in H_2O, 98 ± 3% in D_2O, 70 ± 50% in CMSi, 80 ± 3% in CM4.
[i] 96 ± 3% in H_2O, 90 ± 3% in D_2O, 100 ± 50% in CMSi, 87 ± 3% in CM4.
[j] 99 ± 3% in H_2O, 94 ± 3% in D_2O, 100 ± 50% in CMSi, 91 ± 3% in CM4.
[k] 100 ± 3% in H_2O, 97 ± 3% in D_2O, 100 ± 50% in CMSi, 92 ± 3% in CM4.
[l] 100 ± 3% in H_2O, 99 ± 3% in D_2O, 100 ± 50% in CMSi, 95 ± 3% in CM4.

Table S11. Parameters corresponding to the best fits to the data from *Candida glabrata* bilayers supplemented with Q_{10} before and after addition of *Hs*Δ29DHODH, and after rise, as displayed in Figure 7. Fitting uncertainties are given for the most sensitive contrast.

Layer	τ (Å)	ρ (10^{-6} Å^{-2})	φ (vol%)	σ (Å)	vol% Q_{10}
**Lipid Bilayer**					
Inner lipid heads	9 ± 1	3.0/2.8/2.6/2.4 ± 0.2	46 ± 5	3 ± 1	
Inner lipid chains	13 ± 1	-0.22 ± 0.1	2 ± 2	4 ± 1	
Ubiquinone layer	4 ± 1	0.19 ± 0.1	2 ± 2	1 ± 1	57 ± 14 [a]
Outer lipid chains	13 ± 1	-0.22 ± 0.1	2 ± 2	1 ± 1	
Outer lipid heads	9 ± 1	3.0/2.8/2.6/2.4 ± 0.2	49 ± 5	3 ± 1	
**Bilayer + Protein**					
Layer	τ (Å)	ρ (10^{-6} Å^{-2}) in D_2O/CM4/CMSi/H_2O	φ (vol%)	σ (Å)	vol% DHODH
Inner lipid heads	9 ± 1	3.0/2.8/2.6/2.4 ± 0.2	53 ± 5	5 ± 1	
Inner lipid chains	13 ± 1	-0.22 ± 0.1	10 ± 2	4 ± 1	
Ubiquinone layer	4 ± 1	0.19 ± 0.1	10 ± 2	1 ± 1	
Outer chains + protein	13 ± 1	0.10/0.06/0.02/-0.02 ± 0.1	10 ± 2	1 ± 1	10 ± 5 [a]
Outer heads + protein	8 ± 1	3.0/2.8/2.5/2.3 ± 0.2	50 ± 5	4 ± 1	18 ± 8 [a]
Protein layer 1	40 ± 5	3.0/2.6/2.2/1.8 ± 0.2	70 ± 3 [b]	6 ± 1	30 ± 3 [c]
Protein layer 2	70 ± 15	3.0/2.6/2.2/1.8 ± 0.2	84 ± 3 [d]	10 ± 1	16 ± 3 [c]
Protein layer 3	75 ± 15	3.0/2.6/2.2/1.8 ± 0.2	90 ± 3 [e]	10 ± 1	10 ± 3 [c]
Protein layer 4	75 ± 15	3.0/2.6/2.2/1.8 ± 0.2	91 ± 3 [f]	10 ± 1	9 ± 3 [c]
Protein layer 5	75 ± 15	3.0/2.6/2.2/1.8 ± 0.2	93 ± 3 [g]	10 ± 1	7 ± 3 [c]
Protein layer 6	75 ± 15	3.0/2.6/2.2/1.8 ± 0.2	94 ± 3 [h]	10 ± 1	6 ± 3 [c]
**After Rinse**					
Layer	τ (Å)	ρ (10^{-6} Å^{-2}) in D_2O/CM4/CMSi/H_2O	φ (vol%)	σ (Å)	vol% DHODH
Inner lipid heads	9 ± 1	3.0/2.8/2.6/2.4 ± 0.2	55 ± 5	4 ± 1	
Inner lipid chains	13 ± 1	-0.22 ± 0.1	14 ± 2	4 ± 1	
Ubiquinone layer	4 ± 1	0.19 ± 0.1	14 ± 2	1 ± 1	
Outer chains + protein	12 ± 1	0.10/0.06/0.02/-0.02 ± 0.1	14 ± 2	1 ± 1	10 ± 5 [a]
Outer heads + protein	8 ± 1	3.0/2.8/2.5/2.3 ± 0.2	54 ± 5	4 ± 1	18 ± 8 [a]
Protein layer 1	45 ± 5	3.0/2.6/2.2/1.8 ± 0.2	85 ± 3 [i]	4 ± 1	15 ± 3 [c]
Protein layer 2	75 ± 15	3.0/2.6/2.2/1.8 ± 0.2	86 ± 3 [j]	10 ± 1	14 ± 3 [c]
Protein layer 3	75 ± 15	3.0/2.6/2.2/1.8 ± 0.2	86 ± 3 [k]	10 ± 1	14 ± 3 [c]
Protein layer 4	70 ± 15	3.0/2.6/2.2/1.8 ± 0.2	91 ± 3 [l]	10 ± 1	9 ± 3 [c]
Protein layer 5	75 ± 15	3.0/2.6/2.2/1.8 ± 0.2	93 ± 3 [m]	10 ± 1	7 ± 3 [c]
Protein layer 6	75 ± 15	3.0/2.6/2.2/1.8 ± 0.2	95 ± 3 [n]	10 ± 1	5 ± 3 [c]

[a] Relative to the lipids.
[b] 82 ± 3% in H_2O, 88 ± 3% in D_2O, 82 ± 50% in CMSi, 70 ± 3% in CM4.
[c] Relative to water.
[d] 97 ± 3% in H_2O, 94 ± 3% in D_2O, 100 ± 50% in CMSi, 84 ± 3% in CM4.
[e] 100 ± 3% in H_2O, 95 ± 3% in D_2O, 100 ± 50% in CMSi, 90 ± 3% in CM4.
[f] 100 ± 3% in H_2O, 96 ± 3% in D_2O, 100 ± 50% in CMSi, 91 ± 3% in CM4.
[g] 100 ± 3% in H_2O, 98 ± 3% in D_2O, 100 ± 50% in CMSi, 93 ± 3% in CM4.
[h] 100 ± 3% in H_2O, 99 ± 3% in D_2O, 100 ± 50% in CMSi, 94 ± 3% in CM4.
[i] 91 ± 3% in H_2O, 96 ± 3% in D_2O, 80 ± 50% in CMSi, 85 ± 3% in CM4.
[j] 97 ± 3% in H_2O, 87 ± 3% in D_2O, 100 ± 50% in CMSi, 86 ± 3% in CM4.
[k] 100 ± 3% in H_2O, 92 ± 3% in D_2O, 100 ± 50% in CMSi, 86 ± 3% in CM4.
[l] 100 ± 3% in H_2O, 95 ± 3% in D_2O, 100 ± 50% in CMSi, 91 ± 3% in CM4.
[m] 100 ± 3% in H_2O, 98 ± 3% in D_2O, 100 ± 50% in CMSi, 93 ± 3% in CM4.
[n] 100 ± 3% in H_2O, 100 ± 3% in D_2O, 100 ± 50% in CMSi, 95 ± 3% in CM4.

Table S12. Parameters corresponding to the best fits to the data from bacterial mimic membranes before and after addition of *Ec*DHODH, and after rinse, as displayed in Figure 8. Fitting uncertainties are given for the most sensitive contrast.

**Lipid Bilayer**					
Layer	τ (Å)	ρ (10^{-6} Å^{-2})	φ (vol%)	σ (Å)	
Inner lipid heads	9 ± 1	2.9/2.7/2.6/2.4 ± 0.2	56 ± 8	4 ± 1	
Inner lipid chains	16 ± 1	-0.27 ± 0.1	9 ± 2	6 ± 1	
Outer lipid chains	16 ± 1	-0.27 ± 0.1	9 ± 2	3 ± 1	
Outer lipid heads	9 ± 1	2.9/2.7/2.6/2.4 ± 0.2	56 ± 8	6 ± 1	
**Bilayer + Protein**					
Layer	τ (Å)	ρ (10^{-6} Å^{-2}) in D_2O/CM4/CMSi/H_2O	φ (vol%)	σ (Å)	vol% DHODH
Inner lipid heads	9 ± 1	2.9/2.7/2.6/2.4 ± 0.2	60 ± 8	5 ± 1	
Inner lipid chains	16 ± 1	-0.27 ± 0.1 [a]	16 ± 2 [b]	6 ± 1	
Outer chains + protein	15 ± 1	0.55/0.45/0.35/0.11 ± 0.1	16 ± 2 [b]	4 ± 1	25 ± 6 [c]
Outer heads + protein	8 ± 1	2.9/2.7/2.5/2.3 ± 0.2	62 ± 5	5 ± 1	56 ± 8 [c]
Protein layer 1	40 ± 5	3.0/2.6/2.2/1.8 ± 0.2	63 ± 2 [d]	5 ± 1	37 ± 2 [e]
Protein layer 2	75 ± 15	3.0/2.6/2.2/1.8 ± 0.2	86 ± 3 [f]	5 ± 1	14 ± 3 [e]
Protein layer 3	75 ± 15	3.0/2.6/2.2/1.8 ± 0.2	93 ± 3 [g]	10 ± 1	7 ± 3 [e]
**After Rinse**					
Layer	τ (Å)	ρ (10^{-6} Å^{-2}) in D_2O/CM4/CMSi/H_2O	φ (vol%)	σ (Å)	vol% DHODH
Inner lipid heads	9 ± 1	2.9/2.7/2.6/2.4 ± 0.2	60 ± 8	5 ± 1	
Inner lipid chains	16 ± 1	-0.27 ± 0.1 [a]	19 ± 2 [b]	6 ± 1	
Outer chains + protein	15 ± 1	0.55/0.45/0.35/0.11 ± 0.1	19 ± 2 [b]	4 ± 1	25 ± 6 [c]
Outer heads + protein	8 ± 1	2.9/2.7/2.5/2.3 ± 0.2	66 ± 5	3 ± 1	56 ± 8 [c]
Protein layer 1	40 ± 5	3.0/2.6/2.2/1.8 ± 0.2	66 ± 2 [h]	8 ± 1	34 ± 2 [e]
Protein layer 2	75 ± 15	3.0/2.6/2.2/1.8 ± 0.2	86 ± 3 [i]	8 ± 1	14 ± 3 [e]
Protein layer 3	60 ± 15	3.0/2.6/2.2/1.8 ± 0.2	92 ± 3 [j]	10 ± 1	8 ± 3 [e]
Protein layer 4	60 ± 15	3.0/2.6/2.2/1.8 ± 0.2	98 ± 3 [k]	10 ± 1	2 ± 3 [e]

[a] 0.11 ± 0.1 × 10^{-6} Å^{-2} in H_2O.
[b] 0 ± 2 vol% for H_2O.
[c] Relative to the lipids.
[d] 66 ± 3% in H_2O, 75 ± 50% in CMSi, 70 ± 3% in CM4, 63 ± 3% in D_2O.
[e] Relative to water.
[f] 93 ± 3% in H_2O, 92 ± 50% in CMSi, 93 ± 3% in CM4, 86 ± 3% in D_2O.
[g] 96 ± 3% in H_2O, 97 ± 50% in CMSi, 94 ± 3% in CM4, 93 ± 3% in D_2O.
[h] 74 ± 3% in H_2O, 88 ± 50% in CMSi, 76 ± 3% in CM4, 66 ± 3% in D_2O.
[i] 96 ± 3% in H_2O, 86 ± 50% in CMSi, 90 ± 3% in CM4, 86 ± 3% in D_2O.
[j] 98 ± 3% in H_2O, 90 ± 50% in CMSi, 91 ± 3% in CM4, 92 ± 3% in D_2O.
[k] 100 ± 3% in H_2O, 100 ± 50% in CMSi, 98 ± 3% in CM4, 98 ± 3% in D_2O.

Figure S3. Multiple sequence alignment of Class II DHODHs of which a crystal structure including the α1-α2 microdomain is available. The alignment was done with CLUSTAL OMEGA (1.2.4) [77]. PLAFA: Plasmodium falciparum PDB 6I55; ECOLI: *Escherichia coli* PDB 1F76; SCHMA *Schistosoma mansoni* PDB 6UY4; HUMAN: *Homo sapiens* PDB 2PRM; RAT: *Rattus rattus* PDB 1UUM. The respective UniProt identifiers for the amino acid sequences used in the alignment are given to the left of each row between vertical lines. Amino acid stretches corresponding to α1-α2 microdomain according to the PDB entries are underlined. Cationic amino acid residues in these regions are marked in yellow.

```
tr|Q54A96|Q54A96_PLAFA      MISKLKPQFMFLPKKHILSYCRKDVLNLFEQKFYYTSKRKESNNMKNESLLRLINYNRYY          60
sp|P0A7E1|PYRD_ECOLI        ------------------------------------------------------------          0
tr|G4VFD7|G4VFD7_SCHMA      ------------------------------------------------------------          0
sp|Q02127|PYRD_HUMAN        ------------------------------------------------------------          0
sp|Q63707|PYRD_RAT          ------------------------------------------------------------          0

tr|Q54A96|Q54A96_PLAFA      NKIDSNNYYNGGKILSNDRQYIYSPLCEYKKKINDISSYVSVPFKINIRNLGTSNFVNNK          120
sp|P0A7E1|PYRD_ECOLI        ------------------------------------------------------------          0
tr|G4VFD7|G4VFD7_SCHMA      ------------------------------------------------------------          0
sp|Q02127|PYRD_HUMAN        ------------------------------------------------------------          0
sp|Q63707|PYRD_RAT          ------------------------------------------------------------          0

tr|Q54A96|Q54A96_PLAFA      KDVLDNDYIYENIKKEKSKHKKIIFLLFVSLFGLYGFFESY-NPEFFLYDIFL-KFCLKY          178
sp|P0A7E1|PYRD_ECOLI        ----------------------------------------------MYYPF--VRKALFQ          12
tr|G4VFD7|G4VFD7_SCHMA      -------------MSRIRT-SLEV---LSLGFGLFTAEALYSGNEHFYKDWFLPTARLLV          43
sp|Q02127|PYRD_HUMAN        -------MAWRHLKKRAQD-AVII---LGGGGLLFASYLMATGDERFYAEHLMPTLQGLL          49
sp|Q63707|PYRD_RAT          -------MAWRQLRKRALD-AVII---LGGGGLLFTSYLTATGDDHFYAEYLMPGLQRLL          49

tr|Q54A96|Q54A96_PLAFA      IDGEICHDLFLLLGK---YNILPYDTSNDSIYACTNIKHLDFINPFGVAAGFDKNGVCID          235
sp|P0A7E1|PYRD_ECOLI        LDPERAHEFTFQQLRRITGTPFEALVRQKVPAKPVNCMGLTFKNPLGLAAGLDKDGECID          72
tr|G4VFD7|G4VFD7_SCHMA      RDGETAHNLSVYLAS---YGFIPHKQRNSFPQLKCKVFGLEFDHPIGLAAGFDKDGKAFM          100
sp|Q02127|PYRD_HUMAN        -DPESAHRLAVRFTS---LGLLPRARFQDSDMLEVRVLGHKFRNPVGIAAGFDKHGEAVD          105
sp|Q63707|PYRD_RAT          -DPESAHRLAVRVTS---LGLLPRATFQDSDMLEVKVLGHKFRNPVGIAAGFDKNGEAVD          105
                             * * .* : .         :     :.     .     * :*.*:***:**.* ..

tr|Q54A96|Q54A96_PLAFA      SILKLGFSFIEIGTITPRGQTGNAKPRIFRDVESRSIINSCGFNNMGCDKVTENLILFRK          295
sp|P0A7E1|PYRD_ECOLI        ALGAMGFGSIEIGTVTPRPQPGNDKPRLFRLVDAEGLINRMGFNNLGVDNLVENVKKAHY          132
tr|G4VFD7|G4VFD7_SCHMA      GLLNAGFSHIEVGTVTPNPQLGNARPRIFRWTEKEAVVNRCGFNSDGHDAVYERLKDRPW          160
sp|Q02127|PYRD_HUMAN        GLYKMGFGFVEIGSVTPKPQEGNPRPRVFRLPEDQAVINRYGFNSHGLSVVEHRLRARQQ          165
sp|Q63707|PYRD_RAT          GLYKLGFGFVEVGSVTPQPQEGNPRPRVFRLPEDQAVINRYGFNSHGLSVVEHRLRARQQ          165
                            .:   **. :*:*::**. * ** :**:**  : ..::*  ***. * . : ..:

tr|Q54A96|Q54A96_PLAFA      RQEEDKLLSKHIVGVSIGKNKDT--VNIVDDLKYCINKIGRYADYIAINVSSPNTPGLRD          353
sp|P0A7E1|PYRD_ECOLI        ---------DGVLGINIGKNKDTPVEQGKDDYLICMEKIYAYAGYIAINISSPNTPGLRT          183
tr|G4VFD7|G4VFD7_SCHMA      E-------GRGVIGVNLGCNKTS--ADPTADYVAGVRKFGEVADYLVINVSSPNTPGLRS          211
sp|Q02127|PYRD_HUMAN        KQ-AKLTEDGLPLGVNLGKNKTS--VDAAEDYAEGVRVLGPLADYLVVNVSSPNTAGLRS          222
sp|Q63707|PYRD_RAT          KQ-AQLTADGLPLGINLGKNKTS--EDAAADYAEGVRTLGPLADYLVVNVSSPNTAGLRS          222
                                       :*:.:* ** :   :   *    :. :   *.*:.:*:***** ***

tr|Q54A96|Q54A96_PLAFA      NQEAGKLKNIILSVKEEIDNLEKNNIMNDESTYNEDNKIVEKKNNFNKNNSHMMKDAKDN          413
sp|P0A7E1|PYRD_ECOLI        LQYGEALDDLLTAIKNKQNDLQA-------------------------------------          206
tr|G4VFD7|G4VFD7_SCHMA      LQTKEKLRDLLSKVLAARNQLSK-------------------------------------          234
sp|Q02127|PYRD_HUMAN        LQGKAELRRLLTKVLQERDGLRR-------------------------------------          245
sp|Q63707|PYRD_RAT          LQGKTELRHLLSKVLQERDALKG-------------------------------------          245
                             *    *  ::  :    : *

tr|Q54A96|Q54A96_PLAFA      FLWFNTTKKKPLVFVKLAPDLNQEQKKEIADVL--LETNIDGMIISNTTTQIND------          465
sp|P0A7E1|PYRD_ECOLI        -----MHHKYVPIAVKIAPDLSEEELIQVADSL--VRHNIDGVIATNTTLDRSL------          253
tr|G4VFD7|G4VFD7_SCHMA      ---------KTPILLKISPDENDQNLKDIVEVALDSKTRIDGMIISNTTLTTYEEAVACG          285
sp|Q02127|PYRD_HUMAN        -----VH--RPAVLVKIAPDLTSQDKEDIASVV--KELGIDGLIVTNTTVSRP-------          289
sp|Q63707|PYRD_RAT          -----TR--KPAVLVKIAPDLTAQDKEDIASVA--RELGIDGLIVTNTTVSRP-------          289
                                       : :*::** . ::  ::..     .  ***:* :***

tr|Q54A96|Q54A96_PLAFA      ------IKSFENKKGGVSGAKLKDISTKFICEMYNYTNKQIPIIASGGIFSGLDALEKIE          519
sp|P0A7E1|PYRD_ECOLI        ----VQGMKNCDQTGGLSGRPLQLKSTEIIRRLSLELNGRLPIIGVGGIDSVIAAREKIA          309
tr|G4VFD7|G4VFD7_SCHMA      AAPIPGNNKQNVVYGGLSGRPLFEKSTDCLRKVSALTKGAIPLIGVGGISCGEDALSKLN          345
sp|Q02127|PYRD_HUMAN        ---AGLQGALRSETGGLSGKPLRDLSTQTIREMYALTQGRVPIIGVGGVSSGQDALEKIR          346
sp|Q63707|PYRD_RAT          ---VGLQGALRSETGGLSGKPLRDLSTQTIREMYALTQGRIPIIGVGGVSSGQDALEKIQ          346
                                         **:**  *   **. : .:    :  :*:*. **: .   * .*:

tr|Q54A96|Q54A96_PLAFA      AGASVCQLYSCLVFNGMKSAVQIKRELNHLLYQRGYYNLKEAIGRKHSKS          569
sp|P0A7E1|PYRD_ECOLI        AGASLVQIYSGFIFKGPPLIKEIVTHI-----------------------          336
tr|G4VFD7|G4VFD7_SCHMA      AGASLVQLYTSFVYQGPPVAHKVAREINKLKMTS----------------          379
sp|Q02127|PYRD_HUMAN        AGASLVQLYTALTFWGPPVVGKVKRELEALLKEQGFGGVTDAIGADHRR-          395
sp|Q63707|PYRD_RAT          AGASLVQLYTALIFLGPPVVVRVKRELEALLKERGFTTVTDAIGADHRR-          395
                            ****: *:*: : : *     .:  .:
```
